# Transcriptomic Analysis of Endangered Chinese Salamander: Identification of Immune, Sex and Reproduction-Related Genes and Genetic Markers

**DOI:** 10.1371/journal.pone.0087940

**Published:** 2014-01-31

**Authors:** Rongbo Che, Yuena Sun, Rixin Wang, Tianjun Xu

**Affiliations:** Laboratory of Fish Biogenetics & Immune Evolution, College of Marine Science, Zhejiang Ocean University, Zhoushan, China; Nazarbayev University, Kazakhstan

## Abstract

**Background:**

The Chinese salamander (*Hynobius chinensis*), an endangered amphibian species of salamander endemic to China, has attracted much attention because of its value of studying paleontology evolutionary history and decreasing population size. Despite increasing interest in the *Hynobius chinensis* genome, genomic resources for the species are still very limited. A comprehensive transcriptome of *Hynobius chinensis*, which will provide a resource for genome annotation, candidate genes identification and molecular marker development should be generated to supplement it.

**Principal Findings:**

We performed a *de novo* assembly of *Hynobius chinensis* transcriptome by Illumina sequencing. A total of 148,510 nonredundant unigenes with an average length of approximately 580 bp were obtained. In all, 60,388 (40.66%) unigenes showed homologous matches in at least one database and 33,537 (22.58%) unigenes were annotated by all four databases. In total, 41,553 unigenes were categorized into 62 sub-categories by BLAST2GO search, and 19,468 transcripts were assigned to 140 KEGG pathways. A large number of unigenes involved in immune system, local adaptation, reproduction and sex determination were identified, as well as 31,982 simple sequence repeats (SSRs) and 460,923 putative single nucleotide polymorphisms (SNPs).

**Conclusion:**

This dataset represents the first transcriptome analysis of the Chinese salamander (*Hynobius chinensis*), an endangered species, to be also the first time of hynobiidae. The transcriptome will provide valuable resource for further research in discovery of new genes, protection of population, adaptive evolution and survey of various pathways, as well as development of molecule markers in Chinese salamander; and reference information for closely related species.

## Introduction

The Chinese salamander (*Hynobius chinensis*), a species of salamander in the hynobiidae family, is praised as the “golden key” of studying paleontology evolutionary history and precious “living fossil”. As an endangered species, it has been listed in the China Red Data Book with the National treasure-panda in 1986 and further classified on the International Union for Conservation of Nature and Natural Resources (IUCN) Red List of Threatened Species since 2004. It was first described by Günther in 1889 as *Hynobius chinensis* (*H. chinensis*) from specimens collected in Yichang, Huibei Province [1]. Until it was rediscovered in 2005 no Chinese salamanders had been reported from that area [2].

The species is endemic to China, including Hubei Province (Yichang) of central China, Zhejiang Province (Zhoushan, Wenling, Yiwu, Xiaoshan and Zhenghai counties) and Fujian Province (Chongan and Dehua counties) at the east [3]. Currently, Zhoushan Island holds the densest, perhaps the largest population [4]. Its natural habitats are subtropical or tropical moist lowland forests, rivers, freshwater marshes, freshwater springs, and arable land [5]. It is threatened by habitat destruction and degeneration, in particular due to infrastructure development for human settlement.

Due to the limited sample resource and genomic information, previous studies on Chinese salamander were paid attention to understand its life habits, morphology, biodiversity and population distribution [4], [6], [7]. The Chinese salamander is particularly vulnerable to climate change and diseases caused by persistent organic pollutants, pathogenic microorganism, agricultural and environmental pollutants. The decreasing suitable habitats and increasing disease susceptibility pose particular risks to the local adaptation, immune system and reproduction of *H. chinensis*. Prerequisite conditions to understanding such procedures are knowledge of the genes and pathways involved in local adaptation, immune system, reproductive capacity and sex determination. With respect to molecular markers, which are necessary to support the development of marker assisted selection breeding programs for traits on interest in *H. chinensis* still remain poorly explored. The effective protection of *H. chinensis* population needs comprehensive understanding of the genetic background of the animal populations. With the development of molecular techniques, they have enabled the study of genetic diversity, population structure and genetic variation as well as marker assisted selection breeding, such as simple sequence repeat (SSR) one of the most useful Mendelian markers [8]–[10] and single nucleotide polymorphism (SNP) [11]. Taken together, a fast and cost-efficient approach to exploit important candidate genes involved in local adaptation, immune system and reproduction, as well as molecular markers for *H. chinensis* is required.

Over the last decade, next generation RNA sequencing technologies have provided effective tools for high-throughput sequencing, which has improved the efficiency and speed of gene discovery. Compared to the whole genome sequencing, the next-generation RNA sequencing technologies provide a cost-effective approach to produce transcriptome sequences and molecule markers [12]–[15]. Particularly the Illumina sequencing technology, which is more efficient and inexpensive and can produce more sequences with greater coverage, has made it possible to perform transcriptomic research on many species [16]–[18]. Additionally, a few of amphibians were undertaken a large-scale analysis of transcriptome sequenced by next generation RNA sequencing technologies [19], [20]. In this study, we have used Illumina sequencing to assemble and annotate a transcriptome dataset from *H. chinensis*. A large number of genes involved in the immune system, local adaptation, reproductive capacity and sex determination were identified, the same as a great deal of potential molecular cSSR and SNP markers. This transcriptome dataset provided the first picture of the genomic transcriptional activity of this endangered amphibian species, and moreover, a abundant resource for gene annotation and discovery, identification of genes involved in immune response and adaptive evolution as well as for development molecular markers in the Chinese salamander.

## Results and Discussion

### Illumina Sequencing and Sequence Assembly of *H. chinensis* cDNA

**Figure 1:**
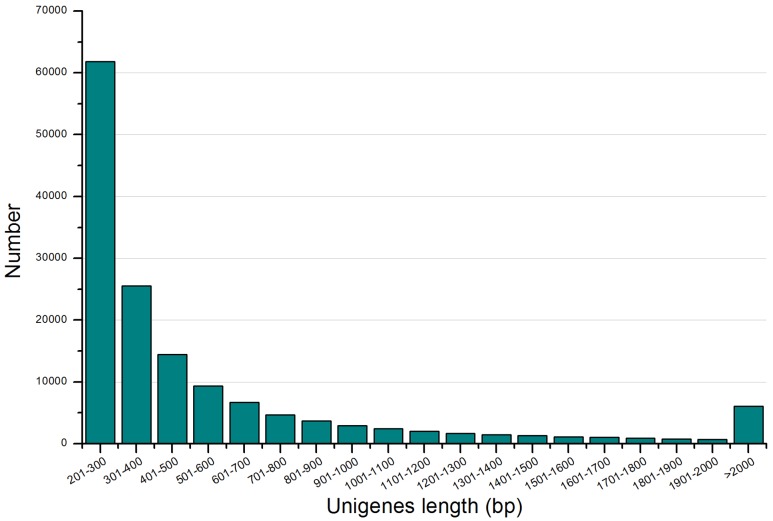
Size distribution of the assembled unigenes.

In order to obtain more detailed information about *H. chinensis* transcriptome, a cDNA library was constructed from RNA isolated from whole animals and sequenced on the Illumina Solexa. As a result, a total of 43,769,857 (98.65%) pair reads with an average length of 77 bases were yielded from 44,367,596 pair reads after quality control by basecalling, which could be used for subsequent splicing analysis (Table 1).Because of there were no assembled and annotated Chinese salamander genomic sequences could be used for transcript assembly; Trinity *de novo* assembler [21] was used to assemble all the trimmed reads with optimized K-mer length of 25. Finally, a total of 148,510 non-redundant unigenes ranging from 201 bp to 21,552 bp with an average length of approximately 580 bp, N50 of 342 bp and a total length of about 86.13 Mb were obtained (Table 1). Among these unigenes, 61,800 unigenes (41.61%) were no more than 300 bp in length, 67,286 unigenes (45.31%) were in the length range of 401 to 1000 bp, and 6,065 unigenes (4.08%) were longer than 2000 bp (Fig. 1).

**Table 1:** Summary of the sequence assembly after Illumina sequencing.

Description	Total Number	Total nucleotides(bp)	Mean length(bp)	N50(bp)
Raw reads	44,367,596 pairs	7,276,285,744	82	
High-quality reads	43,769,857 pairs	6,753,838,879	77	
Unigenes	148,510	86,128,011	580	342
Kmer	25			
High-quality reads percentage	98.65%			
N percentage	0.00%			
Range of unigenes length (bp)	201–21,552			

### Assembly Evaluation and Annotation

**Figure 2:**
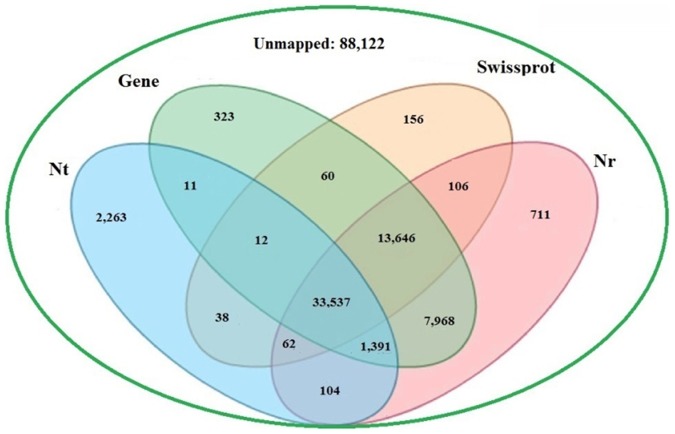
Comparison of the number of unigene annotations obtained from the different databases. The number of unigene annotations hits from the Nt, Nr, Swiss-Prot and Gene databases (E-value ≤1E-5), respectively.

**Figure 3:**
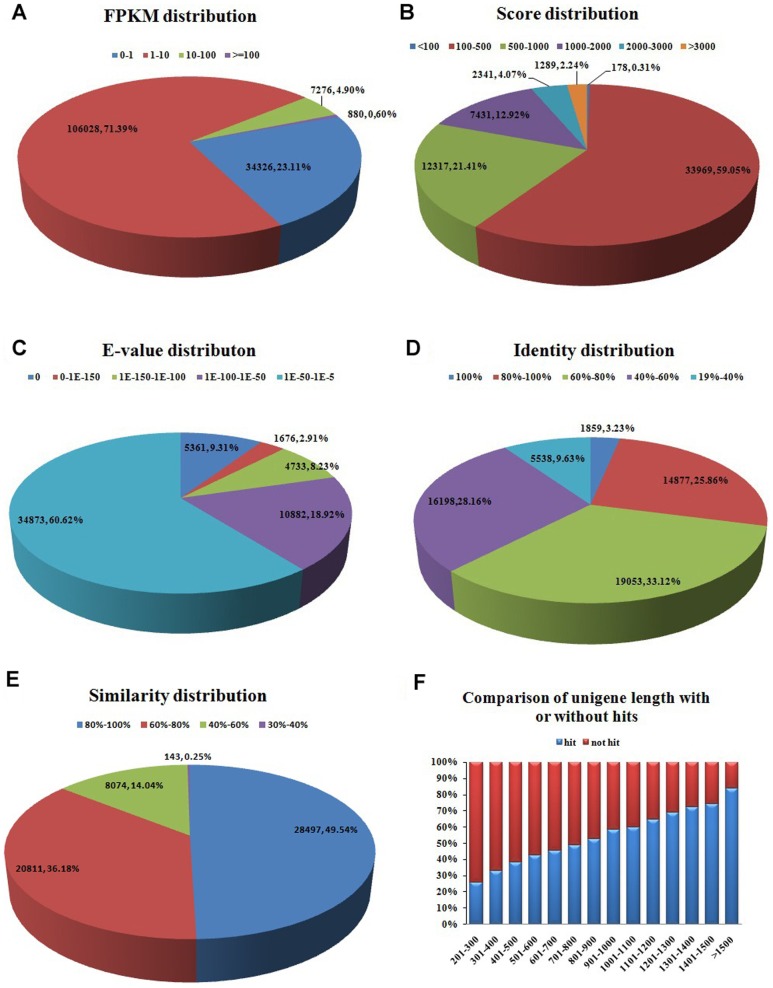
Characteristics of homology search of assembled unigenes against Nr protein database. (**A**) FPKM distribution for each assembled unigene. (**B**) Score distribution of BLAST hits for each unigene with a cutoff E-value of 1E-5. (**C**) E-value distribution of BLAST hits for each unigene with a cutoff E-value of 1E-5. (**D**) Identity distribution of the top BLAST hits for each unigene. (**E**) Similarity distribution of the top BLAST hits for each unigene. (**F**) Length of unigenes with hits compared with those without hits.

**Figure 4:**
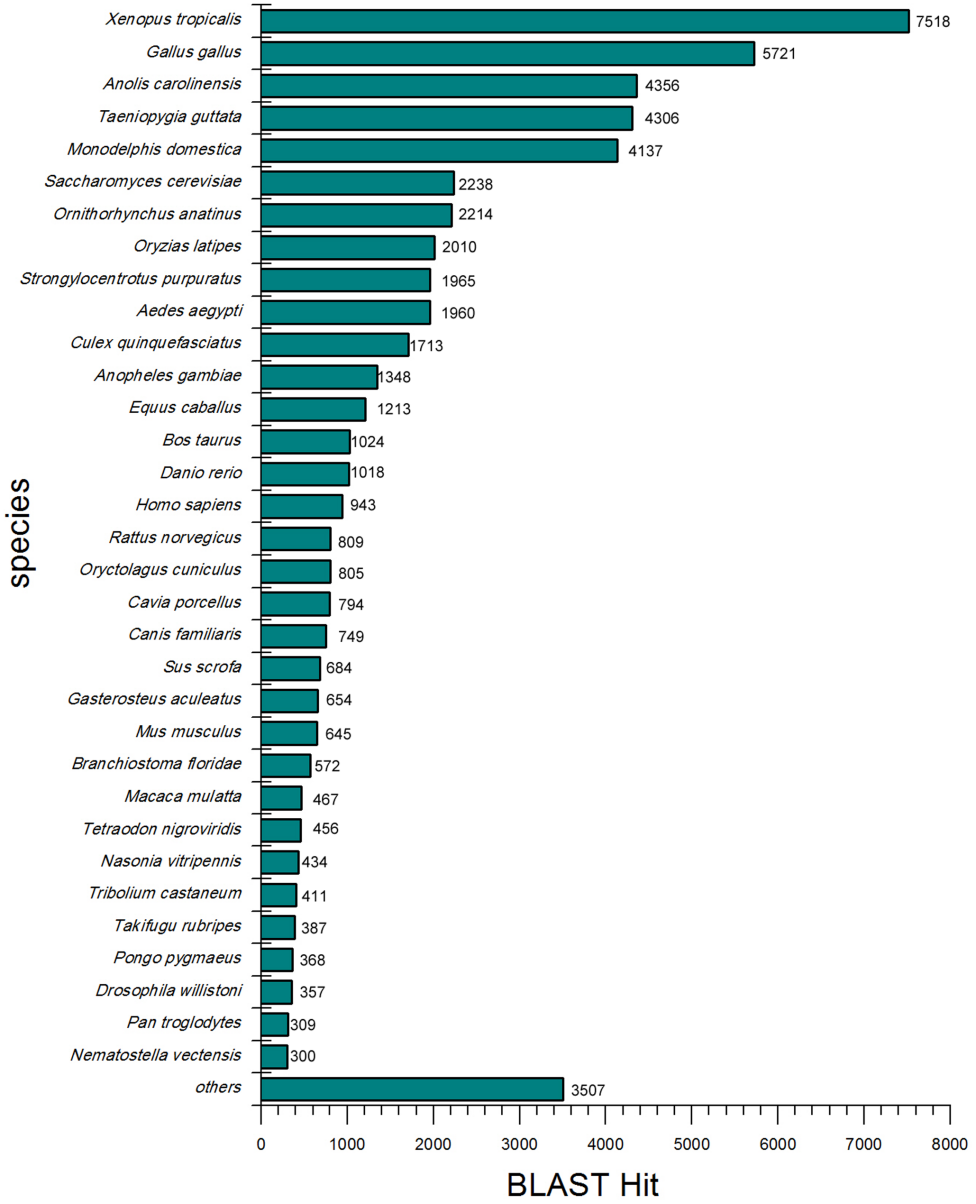
BLASTx top-hit species distribution of unigenes against String database.

To validate and annotate the 148,510 unigenes generated by Trinity, the assembled unigenes were received to BLASTX and BLASTN searches (E-value ≤ 1.0E-5) against public protein databases and nucleotide databases of the National Center for Biotechnology Information (NCBI). The results indicated that out of 148,510 unigenes, 57,525 (38.73%), 47,617 (32.06%), 37,418 (25.20%) and 56,948 (38.35%) unigenes showed similarity with sequences in Nr, Swiss-Prot, Nt and Gene databases, respectively (Fig. 2). Beside these unigenes, 56,549 had homologous sequences both in Nr and Gene databases, 47,315 were concurrently annotated by Nr and Swiss-Prot, 47,183 by Nr, Gene and Swiss-Prot and 34,928 by Nt, Gene and Nr. In all, 60,388 (40.66%) unigenes showed homologous matches in at least one database and 33,537 (22.58%) unigenes were annotated by all four databases, simultaneously (Fig. 2). Moreover, the sequence directions of the resulting unigenes were determined by means of BLAST search and 26,594 unigenes that were not aligned to any of the above databases were determined by using ESTScan software [22], and 57,264 had the same direction in Nr, Gene and Swiss-Prot databases. The unigenes that not be annotated were also effective, but they may putative novel genes or may be too short to show sequence matches or may have undergone a lot of sequence divergence. For evaluation the assembled transcripts, we also research the expression levels and the characteristics of homology search of them. The expression levels were measured using uniquely mapped read pairs. The number of clean reads or fragments of read pairs-end mapped to each annotated unigene was calculated and then normalized to FPKM (Fragments Per Kilobase of gene per Million mapped fragments) metric [23] in a way similar to RPKM [24]. Among 148,510 unigenes, 34,326 (about 23.11%) had the FPKM value of no more than one, 106,028 (approximately71.39%) had the FPKM value between 1 to 10 and 880 (0.60%) had the FPKM value of greater than 100 (Fig. 3A). The result indicated that most transcripts were expressed at low levels. The characteristics of homology search of assembled unigenes were studied on account of the 57,525 unigenes BLAST hits in Nr database for it acting as the most important protein database and there were maximum unigenes annotated by it. The score distribution showed that 1,289 (2.24%) unigenes had scores more than 3,000, and 33,969 (59.05%) had scores falling in between 100 to 500 (Fig. 3B). The E-value distribution revealed that 22,652 unigenes showed significant homology to previously deposited sequences (less than 1.0E-50), and 34,873 ranged from 1.0E-50 to 1.0E-5 (Fig. 3C). Similarly, the identity distribution indicated that 16,736 unigenes with greater 80% identity were found and 33.12% possessing the identity between 60%—80% (Fig. 3D). According to similarity distribution, 49.54% of the assembled unigenes had the similarity of 80%—100%, and 20,811 unigenes obtained the similarity between 60%—80% with the deposited sequences (Fig. 3E). In addition, our result showed that 8,862 (83.77%) unigenes over 1500 bp in length had BLAST matches, while only 25.72% of unigenes shorter than 300 bp did (Fig. 3F), and the longer unigenes were, the higher percentage of BLAST hits were. Indicating that longer unigenes were more likely to obtain BLAST matches in the protein databases, due to the shorter sequences may be too short to show sequence matches or may lack a representative protein domain as reported by [25]–[27]. The BLASTX top-hit species distribution of the 56,393 unigene matched in String database showed the highest isogeny to *Xenopus tropicalis* (7,518 unigenes 13.33%), followed by *Gallus gallus* (5,721 unigenes 10.15%) and *Anolis carolinensis* (4,356 unigenes 7.72%) (Fig. 4).

### Functional Classification by GO and KEGG

**Figure 5:**
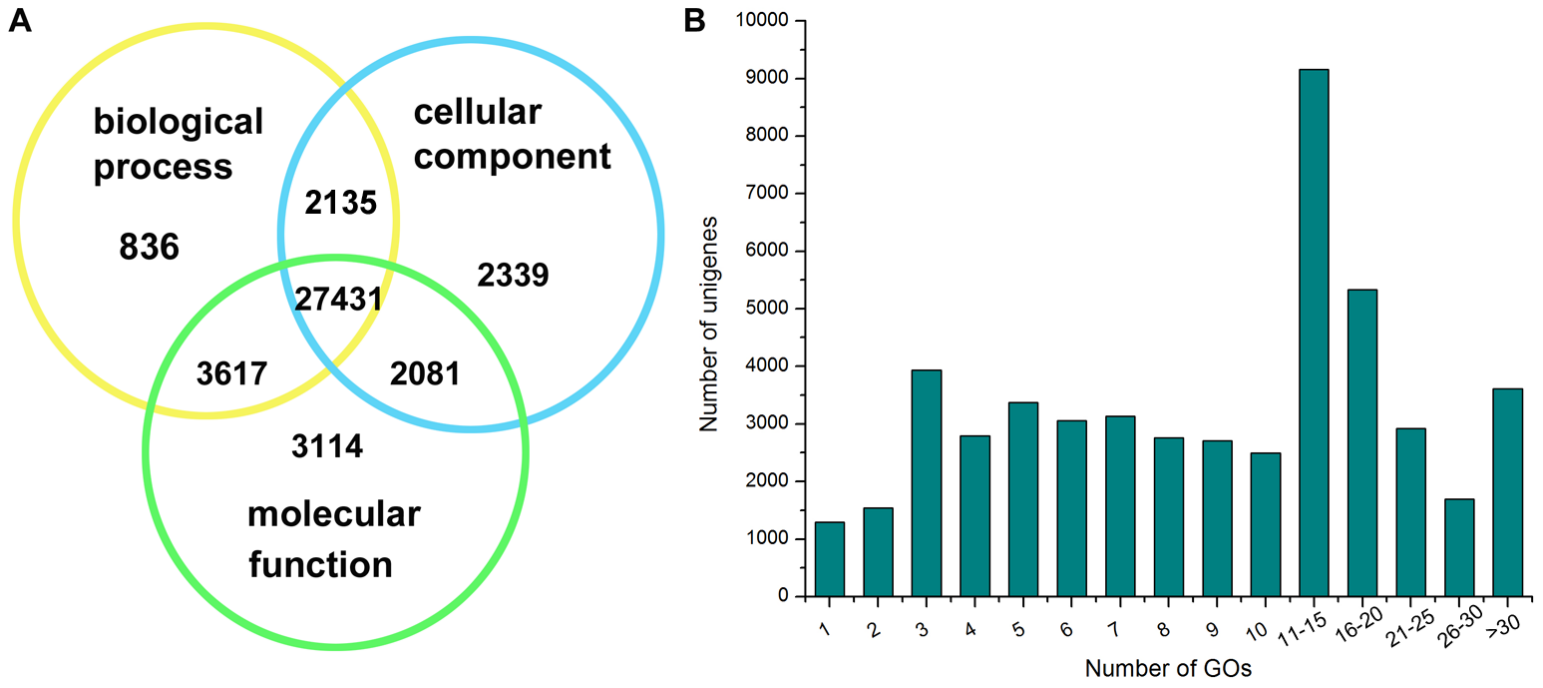
The distribution for assembled unigenes functional classification by Gene Ontology. (**A**) The distribution for assembled unigenes assigned to molecular functions, biological processes and cellular components. (**B**)The number of GO term annotations of each unigene was assigned.

To functionally classify *H. chinensis* unigenes, Gene Ontology (GO), an international standardized gene functional classification system was assigned to each assembled unigene. Based on the 57,525 unigenes that most significant BLASTX hits against Nr database, a total of 41,553 unigenes were categorized into 62 sub-categories under three main ontology (molecular functions, cellular components and biological processes) by Blast2GO[28]. Of these unigenes, 36,243 were assigned to molecular functions as the majority followed by 34,019 to biological processes and 33,986 to cellular components. Additionally, 29,566 (71.19% of the 41,553 unigenes) were both annotated with biological processes and cellular components, 31,048 (74.72%) with biological processes and molecular functions, 29,512 (71.02%) with cellular components and molecular functions, and 27,431 (66.01%) were assigned to all three categories concurrently (Fig. 5A). We also calculated the number of GO term annotations of each unigene was assigned. As shown in Fig. 5B, 1,291 unigenes were assigned only one GO term, 9,158 were assigned 11 to15 GO terms and 3,612 were assigned more than 30 GO term annotations. All of these results indicated that a large fraction of transcripts function differentially and interdependently in *H. chinensis* organism.

**Figure 6:**
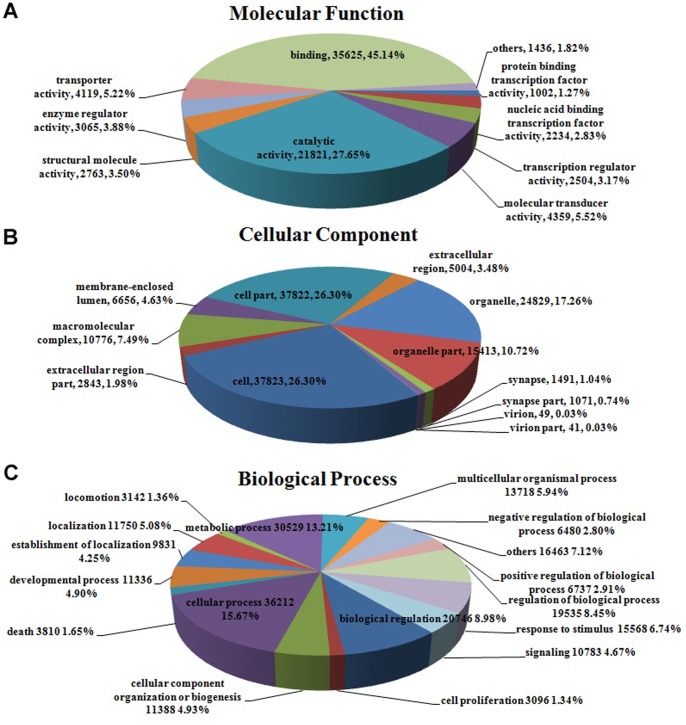
Gene Ontology classifications of assembled unigenes. Unigenes were assigned to three classifications: (**A**) molecular functions (**B**) cellular components and (**C**) biological processes.

Within in molecular function, binding (35,625 unigenes, 45.14%) and catalytic activity (21,821 unigenes, 27.65%) represented the majorities of the category (Fig. 6A), whereas only a few transcripts were assigned to protein tag, morphogen activity, metallochaperone activity and so on (Table S1). Under cellular component category, cell (37,823 unigenes, 26.30%) and cell part (37,822 unigenes, 26.30%) represented the most abundant classification, followed by organelle (24,829 unigenes, 17.26%) and organelle part (15,413 unigenes, 10.72%) (Fig. 6B). For the biological process category, there were 30 sub-categories, cellular process (36,212 unigenes, 15.67%) and metabolic process (30,529 unigenes, 13.21%) were the predominant groups, followed by biological regulation (20,746 unigenes, 8.98%) and regulation of biological process (19,535 unigenes, 8.45%) (Fig. 6C). As *H. chinensis* be an endangered species, the sub-categories, reproductive process and reproduction accounted for a large fraction of unigenes. And a portion of transcripts assigned to immune system process, death, response to stimulus and cell killing sub-categories that are all involved in resistance-related biological processes in the responses to non-biological and biological stimulus were identified (Table S1).

In addition, all unigenes were subjected to a search against the Kyoto Encyclopedia of Genes and Genomes (KEGG) [29] to identify the biological pathways active in *H. chinensis* for further understanding the biological functions and interactions of genes. By performing BLASTX against KEGG database, a total of 19,468 transcripts were assigned to 140 pathways (Table S2). Among them, 10,695 transcripts possessing Enzyme Commission (EC) numbers were assigned to these pathways. These pathways were summarized into 4 main groups: Metabolism (15,106 unigenes), Organismal Systems (147 unigenes), Environmental Information Processing (377 unigenes) and Genetic Information Processing (168 unigenes) (Table S2).

**Figure 7:**
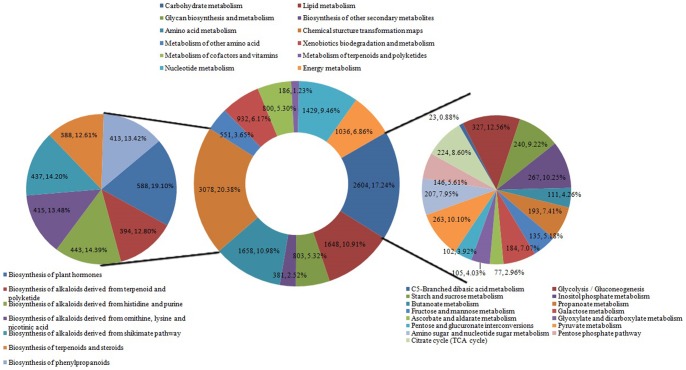
Pathway assignment based on KEGG.

The mapped unigenes represented metabolic pathways of major biomolecules including carbohydrate metabolism, lipid metabolism, amino acid metabolism, chemical structure transformation maps, biosynthesis of secondary metabolites, glycan biosynthesis and metabolism, etc (Fig. 7). In addition, chemical structure transformation maps as the main classification of metabolism group, 3,078 unigenes (about 20.38%) were distributed into 7 pathways consisted of biosynthesis of plant hormones (588 unigenes), terpenoids and steroids (388 unigenes), phenylpropanoids (413 unigenes) and alkaloids derived from terpenoid and polyketide (394 unigenes), histidine and purine (443 unigenes), ornithine, lysine and nicotinic acid (415 unugenes) and shikimate (437 unigenes). Followed by carbohydrate metabolism, 2,604 unigenes (approximately 17.24%) were classified into 15 pathways including glycolysis/gluconeogenesis (327 unigenes), inositol phosphate metabolism (267 unigenes), pyruvate metabolism (263 unigenes), starch and sucrose metabolism (240 unigenes) etc. It was particularly worth mentioning that 140 transcripts were sorted to immune system. The detailed information of other pathways was given in Table S2.

### Important Candidate Genes of *H. chinensis* Transcriptome

For the purpose of further studies of immune system, local adaptation, reproductive capacity and sex determination of *H. chinensis* for it as an endangered breed. We identified full length and partial sequences for a large number of candidate genes associated with these functions according to a keyword search of our BLAST results to the NCBI databases.

**Figure 8:**
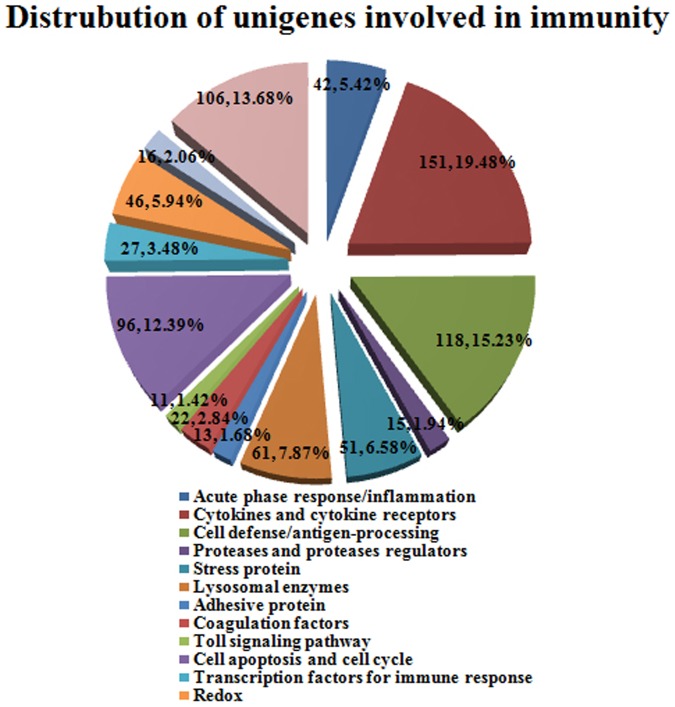
Distribution of unigenes involved in immunity.

By performing this means, a total of 775 transcripts involved in immune function were discovered, including complement component, MHC, Hsp, Cathepsin, Peroxiredoxin, Toll-like receptor, etc (Table S3). All of these immune-relevant genes were classified into 14 groups based on their predicated function, such as cytokines and cytokine receptors (151 unigenes, 19.48%), cell defense/antigen-processing (118 unigenes, 15.23%), cell apoptosis and cell cycle (96 unigenes, 12.39%) and toll signaling pathway (11 unigenes, 1.42%) (Fig. 8). The detailed classification, putative function and matched species of these immune genes were given in Table S4. Major histocompatibility complex (MHC) is the highly polymorphic gene group that widely exists in the vertebrate body closely with immune function [30], [31]. For the MHC genes in amphibians were studied began in the 1970s, Du Pasquier [32] firstly proved the existence of MHC genes and opened the prelude of MHC genes researches in amphibians by the way of MLR, rejection and red cell antigen response. So far, a series of reports on amphibians MHC were, including *Xenopus laevis*, *Xenopus tropicalis*, *Trituruscristatus*, *Ambystoma mexicanum*, *Rana temporaria*, etc. Due to MHC genes play an important effect in immune system of animal, they have been increasingly applied to studies of immunogenetics and protection genetic in amphibian species. The MHC identified in our study could be used to explore the immune defense mechanisms of some diseases and prevent the diseases of *H. chinensis* incipiently as it is an important gene family related to disease resistance, and clarify the phylogenetic relationship of *H. chinensis* with related species as MHC genes possessing cross interspecific polymorphism.

**Figure 9:**
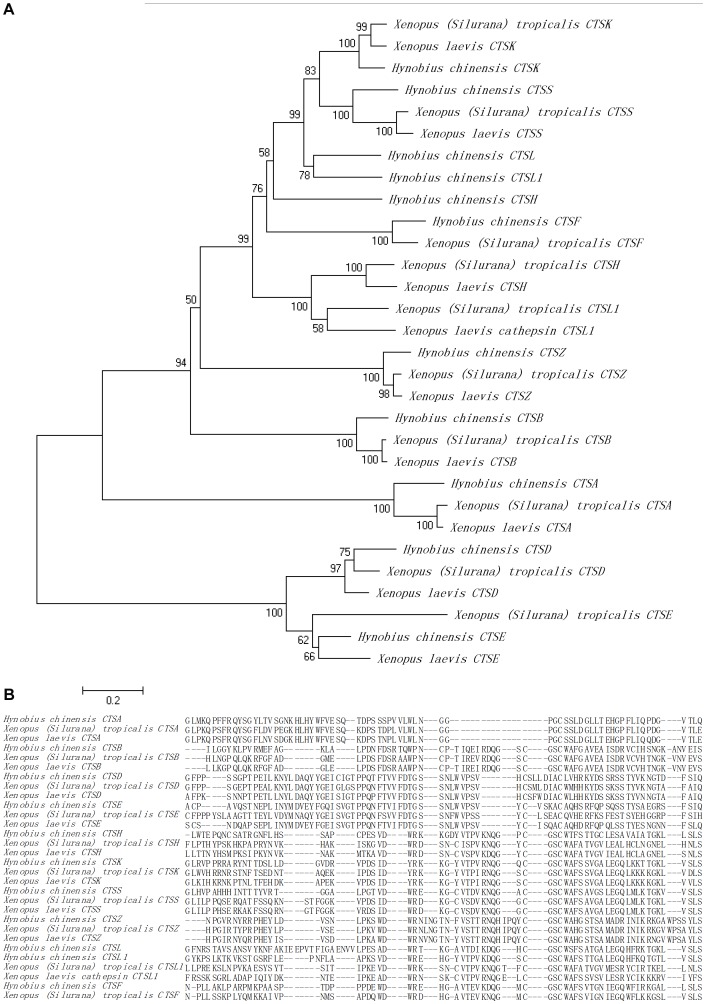
Cathepsins gene phylogeny and amino acid alignment. (**A**) Bootstrap values next to the nodes represent the percentage of 1000 replicate trees supporting the corresponding clade. (**B**) The predicted cathepsin proteins from *H. chinensis* were aligned together with *X. tropicalis* and *X. laevis* cathepsin proteins using MAFFT multiple alignment program.

Cathepsin protein is one of the superfamilies composed of lysosomal proteolytic enzymes that involved in maintaining homeostasis in organisms, which can be categorized into three subgroups according to their amino acid in active sites: cysteine protease, aspartic protease, and serine protease [33], [34]. Particularly, cathepsins play an important role in regulation of antigen presentation and degradation [35]–[37], hormone maturation [38], immune responses [39] and intracellular protein degradation/turn over [40]. In addition to cathepsins (CTS), eleven putative different sequences (CTSA, B, D, E, F, H, K, L, L_1_, S and Z) were identified in *H. chinensis*. Of these sequences were all full-length except for cathepsin L. In order to understand the phylogenetic relationship of cathepsins in *H. chinensis* and other amphibian species, a phylogenetic tree was constructed based on the amino-acid sequences of cathepsins in *H. chinensis* and other 19 cathepsin sequences obtained from another two amphibian species (*Xenopus (Silurana) tropicalis* and *Xenopus laevis*) in publicly available sequence datasets, for there were no more amphibian species cathepsins could be acquired. The overall topology of the tree showed that the *H. chinensis* cathepsin was most similar to the same cathepsin in *X. tropicalis* and *X. laevis* (Fig. 9A), although indicated species-specific gene duplication events for some gene family members (CTSH, L and L_1_). The other cathepsins were grouped in relevant positions with other family members of their respective group (Fig. 9A). The amino acids alignment of the cathepsins with *X. tropicalis* and *X. laevis* indicated highly conserved blocks of amino acids between the same cathepsin and low level of amino acids sequence conservation between different gene family members (Fig. 9B). These cathepsin sequences obtained in *H. chinensis* indicated its high expression level and confirmed one more time of the assembled transcripts.

For *H. chinensis* as a poikilothermal species, a search for genes known to be upregulated in response to temperature found several cold-inducible RNA binding protein (CIRBP) transcripts (Table S3). Several studies showed that its synthesis was induced by low temperature in other species and acted as a translational repressor and regulated by environmental stress in eukaryotic cells [41]. The CIRBP gene was constitutively expressed in male murine germ cell [42], expressed in the gonads early in the sex determining period of *Sphenodon punctatus* [43] and at least three CIRBP homologs are abundantly expressed in *X. laevis* oocytes [44]. These studies indicated that cold-inducible RNA binding protein may be closely link to temperature-dependent sex determination. But the characteristic, function and expression of CIPBP in *H. chinensis* need to be further studied. It is deserve to be mentioned that the heat shock protein (Hsp) genes we identified in immune genes (Hsp40, Hsp60, Hsp70, Hsp90, et al.) also play an important role in temperature-response (Table S3). With sexually dimorphic expression of Hsp27, Hsp70 and Hsp90 observed in American alligator gonadal tissue [45], indicating its potential significance in temperature- dependent sex determination.

**Figure 10:**
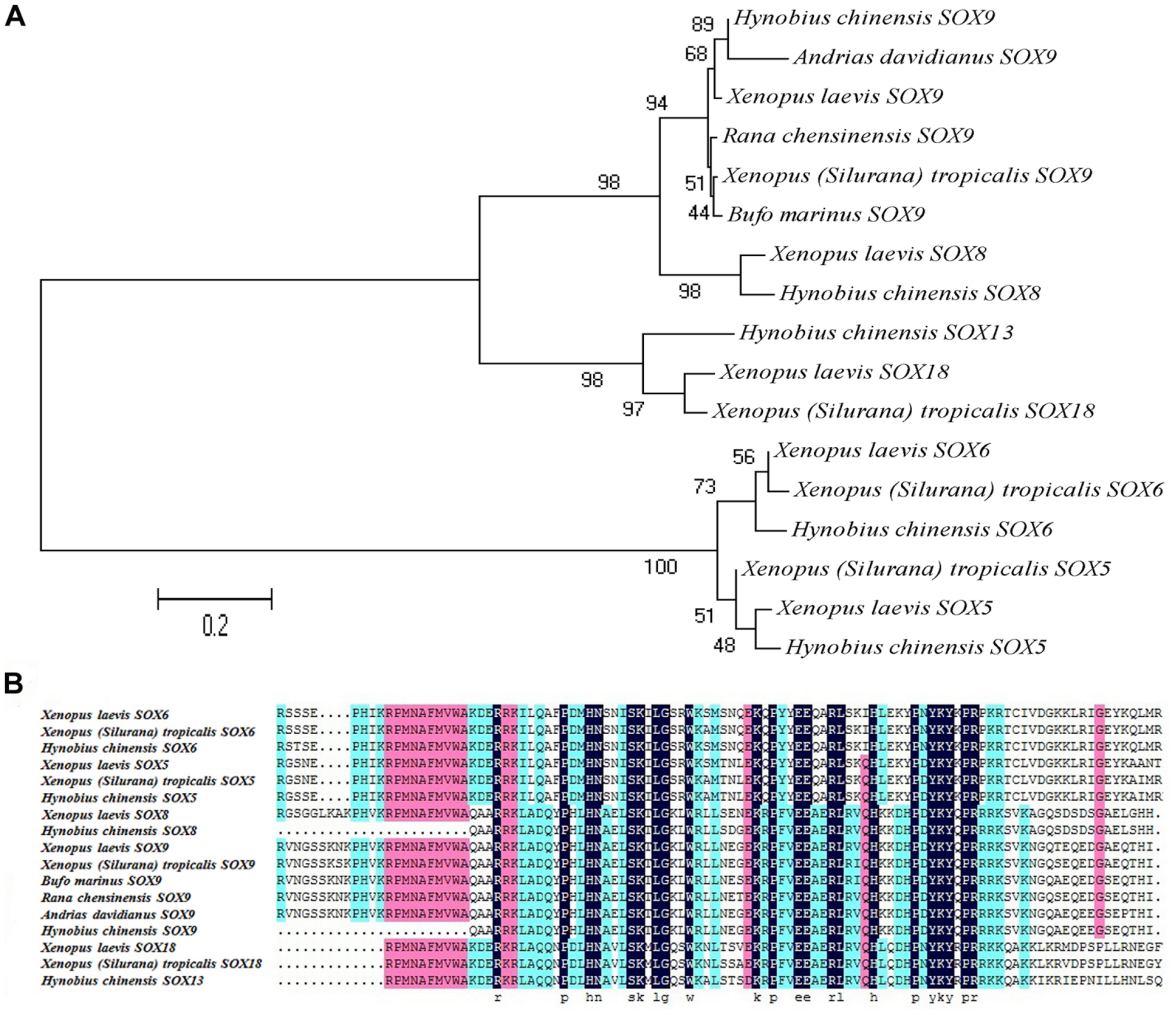
SOXs gene phylogeny and amino acid alignment. (**A**) Bootstrap values next to the nodes represent the percentage of 1000 replicate trees supporting the corresponding clade. (**B**) The predicted SOX proteins from *H. chinensis* were aligned together with *Xenopus (Silurana) tropicalis*, *Xenopus laevis, Bufo marinus, Rana chensinensis* and *Andrias davidianus* SOX proteins using DNAman multiple alignment program.

We also searched for genes known to be involved in sex determination and differentiation, including couple members of sex determination region of Y chromosome-related HMG-box (SOX) family (SOX2-11, 13, 14, 18 et al.), several doublesex and mab-3 related transcription factor (DMRTa1, 2, 3, 5), wilms’tumor suppressor gene-1 (WT1), wingless-type MMTV integration site family, member 4 (WNT4) and forkhead box L2 (FOXL2), but no found of steroidogenic factor 1 (SF1), aromatase, anti-mullerian hormone and dosage-sensitive sex reversal, adrenal hypoplasia critical region, on chromosome X, gene 1 (DAX1) (Table S3). The SOX family of genes has emerged as a pivotal group of genes controlling embryonic development. Since the discovery of SOX genes in 1990 [46], twenty have been found in mice alone, and at least 40 homologues have been identified in insects, nematodes, amphibians, reptiles, birds and a range of mammals including marsupials and humans [47], [48]. The SOX gene family is not only participate in sex determination and differentiation, but also many kinds of the early embryonic development process, such as the development of bone tissue, nervous system, blood cells and crystalline lens [49]-[52]. The main genes involved in sex determination in SOX family are SRY, SOX3, SOX5, SOX6, SOX8, SOX9 and SOX17. The SOX5, SOX6, SOX8, SOX9 and SOX13 of *H. chinensis* were selected to construct a phylogenetic tree with other five amphibian species. The overall topology of the tree showed a close relationship between SOX5 and SOX6, as well as SOX8 and SOX9 (Fig. 10A). The amino acids alignment of the SOX genes showed highly conserved blocks of amino acids between them, particularly the HMG-box region were found in these SOX genes (Fig. 10B). Furthermore, some studies indicated that DMRT [53], FOXL2 [54], DAX1 [55] and SF1 [56], [57] might also play significant roles in sex determination and differentiation in amphibians. The presence of SOX, DMRT, WT1, WNT4 and FOXL2 in our dataset indicated that these might play parts in sex differentiation of *H. chinensis*, but further studies should be processed to elucidate the role of these genes.

The *H. chinensis* was known to be at the risk of extinction, so we used the annotation of our transcripts to identify reproduction-related genes. As a result, at least 33 genes expressed in male or female reproductive tissues or annotated as oocyte-specific, oocyte-related, testis-specific, testis-related and spermatogenesis were found to be defined as reproduction-related genes [58]. And some other unigenes with the same annotation as these reproduction-related genes and other putative reproduction-related genes were listed in Table S5. These genes will provide a valuable genetic resource for further understanding the molecular reproduction mechanisms and the reproductive pathways of *H. chinensis*.

### Development and Characterization of cSSRs and SNPs in Transcriptome

**Figure 11:**
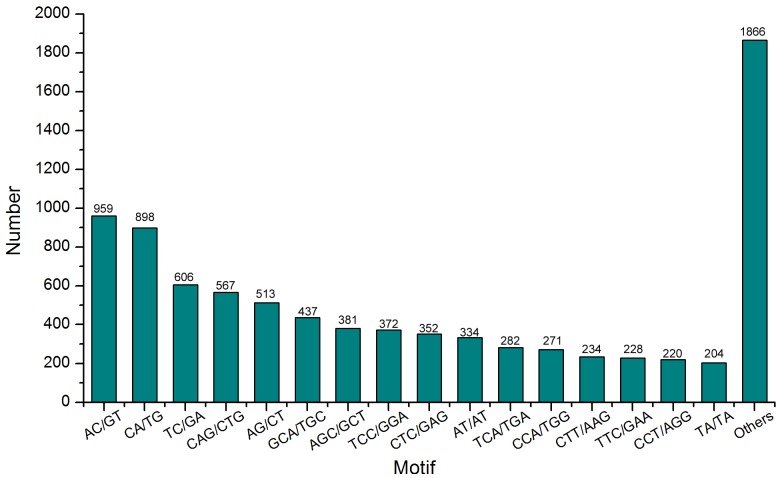
Frequency distribution of cSSRs based on motif sequence types.

Using the MISA Perl script, a total of 24,131 unigenes containing 31,982 cSSRs were identified from 148,510 unigenes, with 5,247 of the sequences containing more than one cSSR. Furthermore, the frequency distribution of these putative cSSRs were further counted and analyzed. In this study, with an average one cSSR locus was existed for about every 2.69 kb of *H. chinensis* unigene sequence. After eliminating the Mono-nucleotide repeats, the motif length more than Hexa-nucleotide repeats and cSSR loci with length less than 10 bp in this study, a total of 8,724 cSSRs were finally obtained. Within the 8,724 cSSRs, the most abundant type of repeat motif was Di-nucleotide repeats (3,525), followed by Tetra- (2,088), Tri- (1,747), Penta- (1,089), and Hexa-nucleotide (275) repeat units (Table 2). Di- to Hexa-nucleotide motifs were also collected on the basis of repeat numbers (Table 2). The potential cSSRs with three tandem reiterations (2,697) were the most common, followed by five tandem repeats (2,140) and six tandem repeats (1,363). These cSSRs contained more than 20 tandem reiterations (3) were rare with a frequency far less than 1%, and all the motifs were Di-repeats (Table 2). Totally, 284 motif sequence types were identified within the searched cSSRs, Di-, Tri-, Tetra-, Penta- and Hexa-nucleotide repeats were 4, 10, 30, 90 and 150 types, respectively. The most abundant repeat motif in our cSSRs was AC/GT (959, 10.99%), followed by CA/TG (898, 10.29%), TC/GA (606, 6.95%) and CAG/CTG (567, 6.50%) (Fig. 11).

**Table 2:** Length distribution of cSSRs based on the number of repeat units.

Repeat numbers	Motif length					Total
	Di	Tri	Tetra	Penta	Hexa	
3			1525	919	253	2697
4		412	408	154	20	994
5	1119	875	131	13	2	2140
6	1042	300	21			1363
7	511	136	1	2		650
8	302	20				322
9	187	2				189
10	212			1		213
11	137		1			138
12	12		1			13
13						
14		1				1
15						
16						
17						
18		1				1
19						
≥20	3					3
Total	3525	1747	2088	1089	275	8724

For further assessing the quality of the putative cSSRs generated in our study and developing new microsatellite markers, 33 primer pairs were designed and synthesized from the randomly selected unique sequences. Of these, 30 primers successfully amplified expected fragments, and 13 of these microsatellite loci were examined showing allelic polymorphism across ten *H. chinensis* individuals. Then 30 individuals of *H. chinensis* from three colonies were used to assess the molecular characterization of the polymorphic loci. The alleles for each locus were ranged from 3 to 6, with an average of 3.85 (Table 3). The observed heterozygosity (*Ho*) and the expected heterozygosity (*He*) ranged from 0.41 to 0.90 with an average of 0.63 and from 0.63 to 0.81 on average of 0.70, respectively. Polymorphism information content (*PIC*) values of per locus varied from 0.54 to 0.77 and all loci were high polymorphic (*PIC*>0.5) with an average of 0.63 (Table 3). Among the thirteen novel loci, only one loci (comp32386_c0_seq1) was found significantly deviated from Hardy–Weinberg equilibrium (HWE) in the sampled population after Bonferroni correction (P<0.0026, adjusted value). Null alleles were more frequently reported at microsatellite loci [59]. Null alleles seemed to be present in low frequency in the *H. chinensis* loci. Only three loci (comp32386_c0_seq1, comp55574_c0_seq1 and comp16428_c0_seq1) were found null alleles examined by Micro-Checker. There were no stuttering errors and no evidence of allelic dropout in any of the loci analyzed by Micro-Checker, and no significant genotypic linkage disequilibrium was found between all pairs of these 13 loci after Bonferroni correction (P<0.0026). None of these 13 sequences was similar to any of the sequences in the GenBank by a homology search using BLASTN program.

**Table 3:** Characteristics of 13 polymorphic cSSRs loci in *H. chinensis*.

Locus	GeneBank accession	Primer sequence	Tm(°C)	Repeat type	Size range/bp	N	Ne	HWE (P)	*PIC*	*Null*	*Ho/He*
											
comp30707_c0_seq1	KC528797	F:TATTCCTTTATTTAATGCTGTC	50	(CT)6	160–164	3	2.65	0.07	0.55	0	0.53/0.63
		R:GAGACGGATTCCCTTGAGTA									
comp5875_c0_seq1	KC528798	F:TCATACACGGATTCATACAGA	58	(TA)7	121–125	3	2.99	0.33	0.59	0	0.63/0.66
		R:TGGAATAGACTTACGAATAAGAG									
comp77539_c0_seq1	KC528799	F: AGCACTAAACCCATCCCAAT	58	(AC)9	100–106	4	3.49	0.39	0.66	0	0.60/0.72
		R: GCAGAGTCTGATGGGTGTATA									
comp5228_c0_seq3	KC528800	F: ATCCAAGTTTGCCGTCAG	58	(AC)16	98–106	3	2.96	0.66	0.59	0	0.69/0.67
		R: AATCCGCCTCCTCGCTCT									
comp7499_c0_seq1	KC528801	F: TCCTGTTAGTCTGAAAAGCC	58	(GA)8	143–153	6	4.98	0.35	0.77	0	0.79/0.81
		R: TCTCACGTAATGATTCTCCA									
comp16428_c0_seq1	KC528802	F: ATGTCTATTGAGTGACTTGCT	58	(TG)8	139–143	3	2.97	0.01	0.59	1	0.41/0.67
		R: ATTGTAATGGTTTGGAGGAT									
comp35785_c0_seq1	KC528803	F: TGTGACAGATGAGAATGAAACT	55	(TG)8	169–177	3	2.92	0.09	0.58	0	0.50/0.67
		R: TAGGGAAGGACAAAGCCA									
comp11916_c0_seq1	KC528794	F: GCACTAGGGCACCCATAA	58	(CT)10	100–104	3	2.60	0.06	0.54	0	0.73/0.63
		R: TTTACGCTCTTTCCCCAA									
comp15080_c0_seq5	KC528805	F: AGATGACAGAGGGGAGATTT	58	(GT)10	159–169	4	2.97	0.64	0.61	0	0.77/0.67
		R: GGCGGTGAGGAGGATGAGCG									
comp8900_c1_seq1	KC528806	F: TCAATGGCATTATCAGTCC	56	(TA)12	143–147	3	2.87	0.81	0.58	0	0.63/0.66
		R: CCCCTTTTACACGGTCAC									
comp55574_c0_seq1	KC528807	F: TACCTGGCTCTGTCCCT	58	(AC)10	238–244	4	3.92	0.01	0.70	1	0.50/0.76
		R: AACCTGCCTCTTTCCC									
comp38566_c0_seq1	KC528808	F: GGAAAATCGCAGACACG	58	(AG)8	237–247	5	4.04	0.22	0.71	0	0.90/0.77
		R: CACATAGGAAATGACTCGG									
comp32386_c0_seq1	KC528809	F: AAGCATTCCAGAAGGCG	58	(TG)7	234–244	6	4.88	0.00*	0.77	1	0.53/0.81
		R: AACGGACCAGGGATACA									
Average						3.85	3.4		0.63		0.63/0.70
								

*N*, number of alleles; *Ne*, effective number of alleles; *H_e_/H_o_*, expected and observed heterozyosity; HWE, Hardy-Weinberg equilibrium; “*” show significant deviation from Hardy-Weinberg equilibrium (p<0.0026).

In addition to cSSRs, by mapping against 148,510 reference unigenes we also identified a total of 460,923 putative single nucleotide polymorphisms (SNPs), wherein 264,449 were transitions and 196,474 were transversions. The number of different transition types (A/G, C/T) was similar, and also a similar number of the four transversion types (A/T, A/C, G/T, C/G) were found (Table 4). These SNPs would also be priority candidates for marker development and should be very useful for further genetic or genomic studies on this species [60]. While, all the potential SNP molecular markers need to be validated to eliminate false positives and sequencing errors. This is the first report of development and validation of a set of cSSR markers and discovery of SNPs in *H. chinensis* by deep transcriptome sequencing using next generation sequencing. The molecular markers will be useful for the further studies of genetic variation, population structure, conservation genetics and diversity analysis as well as molecular assistant breeding of Chinese salamander.

**Table 4:** Distribution of SNPs based on different types.

SNPs	Number	SNPs	Number
Transitions		Transversions	
A<->G	134,216	A<->T	45,578
C<->T	130,233	G<->T	58,061
		C<->G	29,265
		A<->C	63,570
Total	264,449(57.37%)	Total	196,474(42.63%)

## Conclusions

This is the first comprehensive investigation on the transcriptome of *H. chinensis*. In this study, we characterized the transcriptome of this endangered species, and identified a large number of candidate genes involved in immune response, local adaptation, reproduction and sex determination as well as abundant genetic markers (cSSRs and SNPs). The transcriptome data assembled in this study will provide a substantial resource for future studies of gene expression in *H. chinensis* and for annotation of the *H. chinensis* genome, and the candidate genes and molecule markers that will be a useful resource for understanding the molecular mechanisms and constructing genetic linkage maps and researching gene-based association in *H. chinensis*.

## Materials and Methods

### Ethics Statement

This study has been approved by the permission (ZJOU-AWC-10-012) from the Zhejiang Ocean University Animal Welfare Committee.

### Sample Preparation and Illumina Sequencing

Chinese salamanders were obtained from Zhoushan Island (Zhejiang, China), Zhoushan Island is the largest island of Zhoushan Archipelago. Total RNA was extracted from the whole body of four Chinese salamanders using Trizol Reagent (Invitrogen) according to the manufacturer’s instruction. Ahead of cDNA library construction, the total RNA was treated with DNase I, and magnetic beads with Oligo (dT) were used to enrich poly (A) mRNA from it. Then, the purified mRNA was disrupted into short fragments and the double-stranded cDNA was synthesized and subjected to end-repair, add poly (A) and connect with sequencing adapters using Truseq™RNA sample prep Kit (Illumina). The suitable fragments purified by 2% agarose gel electrophoresis (Certified Low Range Ultra Agarose, Bio-Rad) were selected as templates for PCR amplification. Finally, the library was sequenced using Illumina HiSeq™ 2000.

### Assembly and Functional Annotation

By means of base calling the obtained original image data was converted into raw sequencing reads. Among them, the reads with adaptor, repeated reads and low-quality reads (with more than 50% Q≤20 bases) which may affect the assembly and analysis were firstly removed. *De novo* transcriptome assembly was performed on these remaining high-quality reads with a short read assembling program-Trinity [21]. Trinity first combines reads of a certain length that overlap to form longer fragments without gaps, which are called contigs. Trinity allowed us to map the reads back to contigs with the help of paired-end reads, it is possible to identify contigs derived from the same transcript as well as the distances between these contigs. These contigs will be further connected with the sequence clustering software TGICL [61] to obtain sequences that cannot be extended on either end, were defined as unigenes. The FPKM method [24], which was able to eliminate the influence of different gene lengths and sequencing discrepancy on the calculation of gene expression, was used to calculate the unigenes expression. The sequence data were deposited in the NCBI Sequence Read Archive under the accession number of SRA115156. And the assembled sequences have been deposited in the NCBI transcriptome shotgun assembly (TSA) database. This transcriptome shotgun assembly project has been deposited at DDBJ/EMBL/GenBank under the accession GAQK00000000.

All unigenes were searched against the National Center for Biotechnology Information (NCBI) Nr, Swiss-Prot, Nt and Gene databases using BLASTX and BLASTN algorithm with an E-value threshold of 1.0E-5 to determine the sequence directions and protein coding regions. If the results from the different databases conflicted with each other, a priority order of Nt, Nr, Gene and Swiss-Prot was followed when deciding the sequence direction of the unigenes. For unigenes that were not aligned to any of those databases were predicted their coding regions and determined sequence directions by using ESTScan software [22]. Based on Nr annotation, the Blast2GO [28] software was used to get the GO annotation, and then a web tool WEGO [62] was used to obtain the GO functional classification of these annotated unigenes to understand the distribution of gene functions of the species at the macro level. The unigenes were further aligned to Kyoto Encyclopedia of Genes and Genomes (KEGG) pathway database for pathway assignments [29].

### Identification of Important Candidate Genes

The identification of important candidate genes was performed mainly according to a keyword search of our BLAST annotation results to the NCBI databases. A set of keywords, composed by a series of representative immune genes were used to predict immune-related genes based on annotation results. Similarly, the keywords cold-inducible RNA-binding protein and the recognized sex-differentiation genes were used to search for temperature-responsive genes and sex-differentiation genes, respectively. In order to find more genes belonging to functions of immune system, local adaptation, reproductive capacity and sex determination in the transcriptome sequences, the GO term and KEGG pathway information were also used to identify important candidate genes. Especially the immune genes, were detected not only as the description in [63] but also according to the GO categories “response to stimulus” and “immune system process”, and KEGG pathways “immune system” and “immune diseases”, and further classified according to the predicted functions and analyzed based on a comprehensive literature review (Table S4). As for reproduction related genes, the GO categories “reproduction” and “reproductive process” having a direct relationship with reproductive capacity were also used for selecting them.

### Identification and Primer Design of cSSR Markers

MicroSAtellite (MISA, http://pgrc.ipk-gatersleben.de/misa/) [64] was used to identify microsatellites in our unigenes. We searched for Di-, Tri-, Tetra-, Penta- and Hexa- nucleotides repeats with a minimum of five repeat units for Di- and Tri-nucleotides, four for Tetra-nucleotides and three for Penta- and Hexa-nucleotides. Compound repeats were defined as those of ≤10 bp between differently apart microsatellites. Primer pairs flanking the SSR motifs were designed using BatchPrimer3 [65] and synthesized by the company Genscript (Nanjing, China). The 33 pairs of primer sequences used to assess the polymorphism of the putative cSSRs were listed in table S6.

### Primer Validation and Polymorphism Assessment

All of the designed primers were validated by PCR reactions on genomic DNA of Chinese salamander. Each PCR reaction consisted of 1.0 µl of 10× reaction buffer, 0.8 µl dNTPs, 0.6 µl of the forward and reverse primers, 1 µl template genomic DNA and 0.1 µl of *Taq* polymerase (Tiangen, 5U/µl) in a finally 10 µl reaction mixture. And the cycling profile was: denaturation at 95°C for 5 min, followed by 30 cycles of 95°C for 30 sec, 55°C for 30 sec, and extension for 30 sec at 72°C, finally followed with a final extension for 5 min at 72°C, and then holding at 4°C. The polymorphism primers were assessed by ten individuals and then amplified by thirty individuals to examine the genetic characterization. The allele sizes were identified according to pBR322 DNA/MspI molecular weight marker (Tiangen). The program Popgene version 1.32 [66] was used to test for number of alleles per locus (*N*), effected number of alleles (*Ne*), expected (*He*) and observed (*Ho*) heterozygosity and departures from Hardy-Weinberg equilibrium (HWE). Null alleles were examined by Micro-Checker [67]. Polymorphism information content (*PIC*) was analyzed using PIC_CALC and GenAlex6 [68]. Arlequin 3.11 software was used to calculate genotypic linkage disequilibrium between these loci [69]. All results for multiple tests were corrected using Bonferroni correction [70].

### SNP Discovery

Putative single nucleotide polymorphisms (SNPs) were detected using SOAPsnp (http://soap.genomics.org.cn/soapsnp.html) [71]–[73] software by mapping against 148,510 reference unigenes.

## Supporting Information

Table S1 The detail classification of Gene Ontology (GO).
(DOC)

Table S2 Pathway enrichment analysis for the Chinese salamander transcriptome
(XLS)

Table S3 Important candidate genes of *H. chinensis* transcriptome.
(DOC)

Table S4 Putative unigenes related to the immune response.
(XLS)

Table S5 Putative sequences related to reproduction.
(XLS)

Table S6 The 33 pairs of primer sequences used to assess the polymorphism of the putative cSSRs.
(DOC)

## References

[1] Adler K, Zhao EM (1990) Studies on hynobiid salamanders, with description of a new genus. Asiat Herpetol Res 3: 37–45.

[2] Wang X, Wu M, Zhang Y, Wang WJ, Liu MY, et al (2007) On the Re-discovery of *Hynobius chinensis* Günther, 1889 from Type-locality and its Description after 116 Years. Sichuan J Zool 26: 57–58.

[3] Berkeley California (2014) AmphibiaWeb. Available: http://amphibiaweb.org. Accessed 2014 Jan 9.

[4] Ma XM, Gu HQ (1999) Studies on distribution and population size of *Hynobius chinensis* on the Zhoushan Island. Sichuan J Zool 18(3): 107–108.

[5] Gu HQ, Geng BR, Xie FR (2004) *Hynobius chinensis* In: IUCN 2013. IUCN Red List of Threatened Species. Version 2013.2. Available: http://www.iucnredlist.org/details/59092/0. Accessed 2014 Jan 9.

[6] Zeng XM, Fei L, Ye CY, Jiang JP (1997) The karyotypes of three species of genus *Hynobius* and *Salamandrella keyserlingerii* . Zool Res 18(3): 341–345.

[7] Qing LY, Chen Q, Zeng XM, Wang YD (2009) The Karyotype of *Hynobius chinensis* . Sichuan J Zool 44: 125–128.

[8] Jarne P, Lagoda PJL (1996) Microsatellites, from molecules to populations and back. Trends Ecol Evol 11: 424–429. 21237902 10.1016/0169-5347(96)10049-5

[9] Hu JB, Zhou XY, Li JW (2010) Development of novel EST-SSR markers for cucumber (*Cucumis sativus*) and their transferability to related species. Sci Hortic 125: 534–538.

[10] Akfirat FS, Uncuoglu AA (2013) Genetic Diversity of Winter Wheat (*Triticum aestivum L.*) Revealed by SSR Markers. Biochem Genet 51: 223–229. 23274711 10.1007/s10528-012-9557-6

[11] Pritchard VL, Campbell NR, Narum SR, Peacock MM, Garza JC (2013) Discovery and characterization of novel genetic markers for use in the management of Lahontan cutthroat trout (*Oncorhynchus clarkii henshawi*). Mol Ecol Res 13: 276–288. 10.1111/1755-0998.12040 23253773

[12] Chen F, Lee Y, Jiang Y, Wang S, Peatman E, et al (2010) Identification and characterization of full-length cDNAs in channel catfish (*Ictalurus punctatus*) and blue catfish (*Ictalurus furcatus*). PLos One 5: e11546. 20634964 10.1371/journal.pone.0011546 PMC2902525

[13] Hoffman JI, Thorne MAS, Trathan PN, Forcada J (2013) Transcriptome of the dead: characterisation of immune genes and genetic marker development from necropsy samples in a free-ranging marine mammal. BMC Genomics 14: 52. 23347513 10.1186/1471-2164-14-52 PMC3563519

[14] Bai XD, Mamidala P, Rajarapu SP, Jones SC, Mittapalli O (2011) Transcriptomics of the Bed Bug (*Cimex lectularius*). PLos One 6(1): e16336. 21283830 10.1371/journal.pone.0016336 PMC3023805

[15] Bajgain P, Richardson BA, Price JC, Cronn RC, Udall JA (2011) Transcriptome characterization and polymorphism detection between subspecies of big sagebrush (*Artemisia tridentata*). BMC Genomics 12: 370. 21767398 10.1186/1471-2164-12-370 PMC3150299

[16] Li DJ, Deng Z, Qin B, Liu XH, Men ZH (2012) *De novo* assembly and characterization of bark transcriptome using Illumina sequencing and development of EST-SSR markers in rubber tree (*Hevea brasiliensis* Muell. Arg.). BMC Genomics13: 192. 10.1186/1471-2164-13-192 PMC3431226 22607098

[17] Wei WL, Qi XQ, Wang LH, Zhang YX, Hua W, et al (2011) Characterization of the sesame (*Sesamum indicum L.*) global transcriptome using Illumina paired-end sequencing and development of EST-SSR markers. BMC Genomics 12: 451. 21929789 10.1186/1471-2164-12-451 PMC3184296

[18] Sadamoto H, Takahashi H, Okada T, Kenmoku H, Toyota M, et al (2012) *De Novo* Sequencing and Transcriptome Analysis of the Central Nervous System of Mollusc Lymnaea stagnalis by Deep RNA Sequencing. Plos One 7: e42546. 22870333 10.1371/journal.pone.0042546 PMC3411651

[19] Powers TSR, Virk SM, Provencio CT, Serrano EC (2012) Probing the *Xenopus laevis* inner ear transcriptome for biological function. BMC Genomics 13: 225. 22676585 10.1186/1471-2164-13-225 PMC3532188

[20] Yang WZ, Qi Y, Bi K, Fu JZ (2012) Toward understanding the genetic basis of adaptation to high-elevation life in poikilothermic species: A comparative transcriptomic analysis of two ranid frogs, *Rana chensinensis* and *R. kukunoris* . BMC Genomics 13: 588. 23116153 10.1186/1471-2164-13-588 PMC3542248

[21] Grabherr MG, Haas BJ, Yassour M, Levin JZ, Thompson DA, et al (2011) Full-length transcriptome assembly from RNA-Seq data without a reference genome. Nat Biotechnol 29: 644–652. 21572440 10.1038/nbt.1883 PMC3571712

[22] Iseli C, Jongeneel CV, Bucher P (1999) ESTScan: a program for detecting, evaluating, and reconstructing potential coding regions in EST sequences. Proc Int Conf Intell Syst Mol Biol. pp 138–148. 10786296

[23] Trapnell C, Williams BA, Pertea G, Mortazavi A, Kwan G, et al (2010) Transcript assembly and quantification by RNA-Seqreveals unannotated transcripts and isoform switching during cell differentiation. Nat Biotechnol 28: 511–515. 20436464 10.1038/nbt.1621 PMC3146043

[24] Mortazavi A, Williams BA, McCue K, Schaeffer L, Wold B (2008) Mapping and quantifying mammalian transcriptomes by RNA-Seq. Nat Methods 5: 621–628. 18516045 10.1038/nmeth.1226 PMC13303166

[25] Parchman TL, Geist KS, Grahnen JA, Benkman CW, Buerkle CA (2010) Transcriptome sequencing in an ecologically important tree species: assembly, annotation, and marker discovery. BMC Genomics 11: 180. 20233449 10.1186/1471-2164-11-180 PMC2851599

[26] Wang ZY, Fang B, Chen JY, Zhang XJ, Luo ZX, et al (2010) *De novo*assembly and characterization of root transcriptome using Illuminapaired-end sequencing and development of cSSR markers in sweetpotato (*Ipomoea batatas*). BMC Genomics 11: 726. 21182800 10.1186/1471-2164-11-726 PMC3016421

[27] Wang XJ, Xu RH, Wang RL, Liu AZ (2012) Transcriptome analysis of Sacha Inchi (*Plukenetia volubilis L.*) seeds at two developmental stages. BMC Genomics 13: 716. 23256450 10.1186/1471-2164-13-716 PMC3574040

[28] Conesa A, Gotz S, Garcia-Gomez JM, Terol J, Talon M, et al (2005) Blast2GO: a universal tool for annotation, visualization and analysis in functional genomics research. Bioinformatics 21: 3674–3676. 16081474 10.1093/bioinformatics/bti610

[29] Kanehisa M, Goto S, Kawashima S, Okuno Y, Hattori M (2004) The KEGG resource for deciphering the genome. Nucleic Acids Res 32: D277–280. 14681412 10.1093/nar/gkh063 PMC308797

[30] Bjorkman PJ, Saper MA, Samraoui B, Bennett WS, Strominger JL, et al (1987) Structure of the human class I histocompatibility antigen, HLA-A2. Nature 329: 506–512. 3309677 10.1038/329506a0

[31] Edwards SV, Hedrick PW (1998) Evolution and ecology of MHC molecules: from genomics to sexual selection. Trends Ecol Evol 13: 305–311. 21238318 10.1016/s0169-5347(98)01416-5

[32] Hsing LC, Rudensky AY (2005) The lysosomal cysteine proteases in MHC class II antigen presentation. Immunol Rev 207: 229–241. 16181340 10.1111/j.0105-2896.2005.00310.x

[33] Liaudet-Coopman E, Beaujouin M, Derocq D, Garcia M, Glondu-Lassis M, et al (2006) Cathepsin D: newly discovered functions of a longstanding aspartic protease in cancer and apoptosis. Cancer Lett 237: 167–179. 16046058 10.1016/j.canlet.2005.06.007

[34] de Duve C (1983) Lysosomes revisited. Eur J Biochem 137: 391–397. 6319122 10.1111/j.1432-1033.1983.tb07841.x

[35] Honey K, Rudensky AY (2003) Lysosomal cysteine proteases regulate antigen presentation. Nat Rev Immunol 3: 472–482. 12776207 10.1038/nri1110

[36] Liu XZ, Shi G, Cui DL, Wang RX, Xu TJ (2012) Molecular cloning and comprehensive characterization of cathepsin D in the Miiuy croaker *Miichthys miiuy* . Fish Shellfish Immunol 32: 464–468. 22155279 10.1016/j.fsi.2011.11.033

[37] Sun YN, Xu TJ, Wang JX, Cheng YZ, Wang RX (2011) Sequence and expression analysis of cathepsin S gene in the miiuy croaker *Miichthys miiuy* . Fish Physiol Biochem 37: 761–765. 21424529 10.1007/s10695-011-9475-2

[38] Yasothornsrikul S, Greenbaum D, Medzihradszky KF, Toneff T, Bundey R, et al (2003) Cathepsin L in secretory vesicles functions as a prohormone-processing enzyme for production of the enkephalin peptide neurotransmitter. Proc Natl Acad Sci USA 100: 9590–9595. 12869695 10.1073/pnas.1531542100 PMC170962

[39] Dixit AK, Dixit P, Sharma RL (2008) Immunodiagnostic/protective role of Cathepsin L cysteine proteinases secreted by *Fasciola species* . Vet Parasitol 154: 177–184. 18486345 10.1016/j.vetpar.2008.03.017

[40] Bond JS, Butler PE (1987) Intracellular proteases. Annu Rev Biochem 56: 333–364. 3304137 10.1146/annurev.bi.56.070187.002001

[41] De Leeuw F, Zhang T, Wauquier C, Huez G, Kruys V, et al (2007) The cold-inducible RNA-binding protein migrates from the nucleus to cytoplasmic stress granules by a methylation-dependent mechanism and acts as a translational repressor. Exp Cell Res 313: 4130–4144. 17967451 10.1016/j.yexcr.2007.09.017

[42] Nishiyama H, Danno S, Kaneko Y, Itoh K, Yokoi H, et al (1998) Decreased expression of cold-inducible RNA-binding protein (CIRP) in male germ cells at elevated temperature. Am J Pathol 152: 289–296. 9422546 PMC1858111

[43] Rhen T, Schroeder A (2010) Molecular mechanisms of sex determination in reptiles. Sex Dev 4: 16–28. 20145384 10.1159/000282495 PMC2918650

[44] Matsumoto K, Aoki K, Dohmae N, Takio K, Tsujimoto M (2000) CIRP2, a major cytoplasmic RNA-binding protein in Xenopus oocytes. Nucleic Acids Res 28: 4689–4697. 11095679 10.1093/nar/28.23.4689 PMC115157

[45] Kohno S, Katsu Y, Urushitani H, Ohta Y, Iguchi T, et al (2010) Potential contributions of heat shock proteins to temperature-dependent sex determination in the American alligator. Sex Dev 4: 73–87. 19940440 10.1159/000260374 PMC2855287

[46] Gubbay J, Collignon J, Koopman P, Capel B, Economou A, et al (1990) A gene mapping to the sex-determining region of the mouse Y chromosome is a member of a novel family of embryonically expressed genes. Nature 346: 245–250. 2374589 10.1038/346245a0

[47] Wegner M (1999) From head to toes: the multiple facets of Sox proteins. Nucleic Acids Res 27: 1409–1420. 10037800 10.1093/nar/27.6.1409 PMC148332

[48] Han F, Wang ZJ, Wu FR, Liu ZH, Huang BF, et al (2010) Characterization, phylogeny, aherative splicing and expression of Sox30 gene. BMC Mol Biol 11(98): 1471–1482. 10.1186/1471-2199-11-98 PMC3004900 21143990

[49] Pevny LH, Lovell-Badge R (1997) Sox genes find their feet. Curr Opin Genet Dev 7: 338–344. 9229109 10.1016/s0959-437x(97)80147-5

[50] Akiyama H, Chaboissier MC, Martin JF, Schedl A, de Crombrugghe B (2002) The transcription factor Sox9 has essential roles in successive steps of the chondrocyte differentiation pathway and is required for expression of Sox5 and Sox6. Genes Dev 16: 2813–2828. 12414734 10.1101/gad.1017802 PMC187468

[51] Jay P, Goze C, Marsollier C, Taviaux S, Hardelin JP, et al (1995) The human SOX11 gene: cloning, chromosomal assignment and tissue expression. Genomics 29: 541–545. 8666406 10.1006/geno.1995.9970

[52] Wright E, Hargrave MR, Christiansen J, Cooper L, Kun J, et al (1995) The Sryrelated gene Sox9 is expressed during chondrogenesis in mouse embryos. Nat Genet 9: 15–20. 7704017 10.1038/ng0195-15

[53] Shibata K, Takase M, Nakamura M (2002) The Dmrt1 Expression in Sex- Reversed Gonads of Amphibians. Gen Comp Endocrinol 127: 232–241. 12225764 10.1016/s0016-6480(02)00039-4

[54] Oshima Y, Uno Y, Matsuda Y, Kobayashi T, Nakamura M (2008) Molecular cloning and gene expression of Foxl2 in the frog Rana rugosa. Gen Comp Endocrinol 159: 170–177. 18805419 10.1016/j.ygcen.2008.08.013

[55] Sugita J, Takase M, Nakamura M (2001) Expression of Dax-1 during gonadal development of the frog. Gene 280: 67–74. 11738819 10.1016/s0378-1119(01)00739-9

[56] Mayer LP, Overstreet SL, Dyer CA, Propper CR (2002) Sexually dimorphic expression of steroidogenic factor 1 (SF-1) in developing gonads of the American bullfrog, Rana catesbeiana. Gen Comp Endocrinol 127: 40–47. 12161200 10.1016/s0016-6480(02)00019-9

[57] Domenice S, Correa RV, Costa EM, Nishi MY, Vilain E, et al (2004) Mutations in the SRY, DAX1, SF1 and WNT4 genes in Brazilian sex-reversed patients. Braz J Med Biol Res 37: 145–150. 14689056 10.1590/s0100-879x2004000100020

[58] Haerty W, Jagadeeshan S, Kulathinal RJ, Wong A, Ravi Ram K, et al (2007) Evolution in the fast lane: rapidly evolving sex-related genes in *Drosophila* . Genetics 177: 1321–1335. 18039869 10.1534/genetics.107.078865 PMC2147986

[59] Callen DF, Thompson AD, Shen Y, Phillips HA, Richards RI, et al (1993) Incidence and origin of ‘‘null’’ alleles in the (AC)n microsatellite markers. Am J Hum Gene 52: 922–927. PMC1682051 8488841

[60] Hou R, Bao ZM, Wang S, Su HL, Li Y, et al (2011) Transcriptome sequencing and *de novo* analysis for Yesso scallop (*Patinopecten yessoensis*) using 454 GS FLX. PLos One 6: e21560. 21720557 10.1371/journal.pone.0021560 PMC3123371

[61] Pertea G, Huang X, Liang F, Antonescu V, Sultana R, et al (2003) TIGR Gene Indices clustering tools (TGICL): a software system for fast clustering of large EST datasets. Bioinformatics 19: 651–652. 12651724 10.1093/bioinformatics/btg034

[62] Ye J, Fang L, Zheng HK, Zhang Y, Chen J, et al (2006) WEGO: a web tool for plotting GO annotations. Nucleic Acids Res 34: W293–297. 16845012 10.1093/nar/gkl031 PMC1538768

[63] Xu TJ, Meng FX, Sun YN, Shi G, Wang RX (2010) Identification of immune genes of the miiuy croaker (*Miichthys miiuy*) by sequencing and bioinformatic analysis of ESTs. Fish Shellfish Immunol 29: 1099–1105. 20801222 10.1016/j.fsi.2010.08.013

[64] Thiel T, Michalek W, Varshney R, Graner A (2004) Exploiting EST databases for the development and characterization of gene-derived SSR-markers in barley (*Hordeum vulgare L.*). Theor Appl Genet 106: 411–422. 10.1007/s00122-002-1031-0 12589540

[65] Rozen S, Skaletsky H (2000) Primer3 on the WWW for general users and for biologist programmers. Methods Mol Biol 132: 365–386. 10547847 10.1385/1-59259-192-2:365

[66] Yeh FC, Boyle TJB (1997) Population genetic analysis of co-dominant and dominant markers and quantitative traits. Belg J Bot 129: 157.

[67] Van Oosterhout C, Hutchinson WF, Wills DPM, Shipley P (2004) MICRO-CHECKER: software for identifying and correcting genotyping errors in microsatellite data. Mol Ecol Notes 4: 535–538.

[68] Peakall R, Smouse PE (2006) GENALEX 6: genetic analysis in Excel. Population genetic software for teaching and research. Mol Ecol Notes 6: 288–295. 10.1093/bioinformatics/bts460 PMC3463245 22820204

[69] Schneider S, Roessli D, Excoffier L (2000) Arlequin: A software for population genetics data analysis. 2.000 ed. Geneva: Genetics and Biometry Lab, Dept. of Anthropology, University of Geneva.

[70] Rice WR (1989) Analyzing tables of statistical tests. Evol 43: 223–225. 10.1111/j.1558-5646.1989.tb04220.x 28568501

[71] Li R, Li Y, Fang X, Yang H, Wang J, et al (2009) SNP detection for massively parallel whole-genome resequencing. Genome Res 19: 1124–1132. 19420381 10.1101/gr.088013.108 PMC2694485

[72] Du Y, Jiang H, Chen Y, Li C, Zhao MR, et al (2012) Comprehensive evaluation of SNP identification with the Restriction Enzyme-based Reduced Representation Library (RRL) method. BMC Genomics 13: 77. 22340203 10.1186/1471-2164-13-77 PMC3305556

[73] Langmead B, Schatz MC, Lin J, Pop M, Salzberg SL (2009) Searching for SNPs with cloud computing. Genome Biol 10: R134. 19930550 10.1186/gb-2009-10-11-r134 PMC3091327

