# Supplementary material for: Transcriptomic Analysis of Endangered Chinese Salamander: Identification of Immune, Sex and Reproduction-Related Genes and Genetic Markers
Source: PLoS One. 2014 Jan 31;9(1):e87940. doi: 10.1371/journal.pone.0087940 (PMC3909259; doi:10.1371/journal.pone.0087940)
Supplement: Table S1 — The detail classification of Gene Ontology (GO). (DOC) [file pone.0087940.s001.doc]

**Table S2.** The detail classification of Gene Ontology (GO).

| **GO classification** | **Number** |
| --- | --- |
|  |  |
| **Molecular Function** |  |
| protein tag | 5 |
| morphogen activity | 6 |
| metallochaperone activity | 17 |
| chemoattractant activity | 23 |
| nutrient reservoir activity | 32 |
| chemorepellent activity | 33 |
| translation regulator activity | 48 |
| receptor regulator activity | 60 |
| antioxidant activity | 194 |
| channel regulator activity | 199 |
| electron carrier activity | 819 |
| protein binding transcription factor activity | 1002 |
| nucleic acid binding transcription factor activity | 2234 |
| transcription regulator activity | 2504 |
| structural molecule activity | 2763 |
| enzyme regulator activity | 3065 |
| transporter activity | 4119 |
| molecular transducer activity | 4359 |
| catalytic activity | 21821 |
| binding | 35625 |
| **Cellular Component** |  |
| virion part | 41 |
| virion | 49 |
| synapse part | 1071 |
| synapse | 1491 |
| extracellular region part | 2843 |
| extracellular region | 5004 |
| membrane-enclosed lumen | 6656 |
| macromolecular complex | 10776 |
| organelle part | 15413 |
| organelle | 24829 |
| cell part | 37822 |
| cell | 37823 |
| **Biological Process** |  |
| nitrogen utilization | 1 |
| sugar utilization | 1 |
| carbohydrate utilization | 3 |
| carbon utilization | 4 |
| cell killing | 123 |
| pigmentation | 288 |
| rhythmic process | 408 |
| viral reproduction | 785 |
| growth | 1842 |
| multi-organism process | 2153 |
| reproductive process | 2480 |
| reproduction | 2515 |
| biological adhesion | 2870 |
| immune system process | 2990 |
| cell proliferation | 3096 |
| locomotion | 3142 |
| death | 3810 |
| negative regulation of biological process | 6480 |
| positive regulation of biological process | 6737 |
| establishment of localization | 9831 |
| signaling | 10783 |
| developmental process | 11336 |
| cellular component organization or biogenesis | 11388 |
| localization | 11750 |
| multicellular organismal process | 13718 |
| response to stimulus | 15568 |
| regulation of biological process | 19535 |
| biological regulation | 20746 |
| metabolic process | 30529 |
| cellular process | 36212 |
