# Supplementary material for: Transcriptomic Analysis of Endangered Chinese Salamander: Identification of Immune, Sex and Reproduction-Related Genes and Genetic Markers
Source: PLoS One. 2014 Jan 31;9(1):e87940. doi: 10.1371/journal.pone.0087940 (PMC3909259; doi:10.1371/journal.pone.0087940)
Supplement: Table S3 — Important candidate genes of H. chinensis transcriptome. (DOC) [file pone.0087940.s003.doc]

**Table S3.** Candidate genes for immune function, local adaptation, sex determination and reproductive capacity in *H. chinensis* transcriptome.

| **Gene** | **Transcript ID** | **Transcript length** | **Match species** | **Match identity** | **Match accession** |
| --- | --- | --- | --- | --- | --- |
|
| **Immune genes** |  |  |  |  |  |
| Complement component C6 | comp83265_c0_seq1 | 298 | *Gallus gallus* | 54% | NP_001138321.1 |
| Complement component | comp17446_c0_seq1 | 793 | *Aedes aegypti* | 63% | XP_001661410.1 |
| Complement component 7 | comp6278_c1_seq1 | 2844 | *Gallus gallus* | 58% | ADY17228.1 |
| Complement component 2 | comp6731_c0_seq2 | 906 | *Xenopus laevis* | 46% | NP_001116166.1 |
| MHC class II alpha chain | comp61329_c0_seq2 | 313 | *Caiman crocodilus* | 66% | AAF99282.2 |
| MHC class IA alpha chain | comp23480_c0_seq1 | 629 | *Ambystoma mexicanum* | 59% | AAC60108.1 |
| MHC class I heavy chain | comp23480_c0_seq7 | 215 | *Ambystoma mexicanum* | 60% | AAD44914.1 |
| MHC class II beta chain | comp11693_c0_seq5 | 262 | *Ambystoma mexicanum* | 62% | AAG42328.1 |
| Heat shock protein 40 | comp105101_c0_seq1 | 203 | *Locusta migratoria* | 61% | ABC84495.1 |
| Heat shock protein 70 form 2 | comp61469_c0_seq1 | 760 | *Paralvinella grasslei* | 75% | ABU63809.1 |
| Small heat shock protein | comp113930_c0_seq1 | 206 | *Belgica antarctica* | 62% | ABF01017.1 |
| Heat shock factor | comp90007_c0_seq1 | 243 | *Polypedilum vanderplanki* | 60% | ADM13379.1 |
| Heat shock protein gp96 | comp127920_c0_seq1 | 231 | *Strongylocentrotus purpuratus* | 76% | NP_999808.1 |
| Hsp78p | comp43163_c0_seq1 | 2881 | *Saccharomyces cerevisiae* | 98% | NP_010544.1 |
| Small heat shock protein 20 | comp29932_c0_seq1 | 329 | *Polypedilum vanderplanki* | 72% | ADM13384.1 |
| Heat shock protein 60 | comp57832_c0_seq1 | 237 | *Polypedilum vanderplanki* | 89% | ADM13383.1 |
| Hsp10p | comp26464_c0_seq1 | 547 | *Saccharomyces cerevisiae* | 100% | NP_014663.1 |
| Hsp104p | comp17268_c0_seq1 | 2853 | *Saccharomyces cerevisiae* | 99% | NP_013074.1 |
| Hsp12p | comp8526_c0_seq1 | 607 | *Saccharomyces cerevisiae* | 100% | NP_116640.1 |
| Heat shock 90 kDa protein | comp69862_c0_seq1 | 246 | *Priapulus caudatus* | 86% | ABB29634.1 |
| Heat shock 22kDa protein 8 | comp2598_c0_seq1 | 1727 | *Xenopus laevis* | 74% | NP_001079782.1 |
| Heat shock protein 47 | comp2672_c0_seq1 | 3441 | *Alligator mississippiensis* | 56% | BAF94140.1 |
| HSP70 | comp126563_c0_seq1 | 307 | *Chironomus tentans* | 90% | AAN85117.1 |
| Hsp60 protein, putative | comp123265_c0_seq1 | 257 | *Pediculus humanus corporis* | 79% | XP_002427863.1 |
| Hsp90 | comp87866_c0_seq1 | 226 | *Bemisia tabaci* | 63% | ADO14474.1 |
| Cathepsin A | comp1240_c0_seq1 | 1979 | *Xenopus laevis* | 70% | NP_001088109.1 |
| Cathepsin B | comp1249_c0_seq1 | 1296 | *Taeniopygia guttata* | 77% | XP_002186710.1 |
| Cathepsin D | comp1651_c0_seq1 | 1947 | *Hynobius leechii* | 91% | AAD33219.1 |
| Cathepsin E-A precursor | comp2872_c0_seq1 | 1625 | *Xenopus laevis* | 71% | NP_001079043.1 |
| Cathepsin F | comp22573_c0_seq1 | 1421 | *Xenopus (Silurana) tropicalis* | 73% | NP_001106423.1 |
| Cathepsin H | comp3327_c0_seq5 | 1399 | *Gallus gallus* | 72% | AEC13302.1 |
| Cathepsin K | comp1967_c0_seq1 | 2373 | *Sus scrofa* | 76% | NP_999467.1 |
| Cathepsin L | comp6709_c0_seq1 | 1083 | *Aedes aegypti* | 67% | XP_001655999.1 |
| Cathepsin L1 | comp1246_c0_seq2 | 1190 | *Xenopus (Silurana) tropicalis* | 55% | NP_001015747.1 |
| Cathepsin S | comp6891_c0_seq1 | 1266 | *Monodelphis domestica* | 71% | XP_001370975.2 |
| Cathepsin Z | comp1846_c0_seq1 | 1309 | *Xenopus laevis* | 85% | NP_001088101.1 |
| Peroxiredoxins, prx-1, prx-2, prx-3 | comp66124_c0_seq1 | 309 | *Aedes aegypti* | 86% | XP_001663718.1 |
| Peroxiredoxin 1 | comp131054_c0_seq1 | 221 | *Harpegnathos saltator* | 81% | EFN81425.1 |
| Peroxiredoxin 3 | comp1420_c0_seq1 | 1054 | *Rattus norvegicus* | 72% | EDL94585.1 |
| Peroxiredoxin 4 | comp1384_c0_seq1 | 1068 | *Xenopus laevis* | 92% | NP_001085918.1 |
| Peroxiredoxin 6 | comp1178_c0_seq1 | 1545 | *Xenopus laevis* | 80% | NP_001084316.1 |
| Toll-like receptor 1 type 2 | comp56028_c0_seq1 | 726 | *Anas platyrhynchos* | 52% | ACS92622.1 |
| Toll-like receptor 3 | comp49000_c0_seq1 | 578 | *Cavia porcellus* | 54% | NP_001166500.1 |
| Toll-like receptor 10 | comp87180_c0_seq1 | 213 | *Cervus nippon* | 53% | ADZ17131.1 |
| PREDICTED: toll-like receptor 5-like | comp80462_c0_seq1 | 416 | *Xenopus (Silurana) tropicalis* | 71% | XP_002940742.1 |
| Toll-like receptor 22b | comp32569_c0_seq1 | 1073 | *Salmo salar* | 60% | CAR62394.1 |
| **Temperature-responsive genes** |  |  |  |  |  |
| Cold-inducible RNA-binding protein | comp33_c0_seq1 | 974 | *Salmo salar* | 72% | ACI33784.1 |
| CIRBP mRNA for cold-inducible RNA binding protein | comp56_c0_seq1 | 1164 | *Alligator mississippiensis* | 82% | AB306286.1 |
| CIRBP mRNA for cold-inducible RNA binding protein | comp158_c0_seq1 | 303 | *Alligator mississippiensis* | 86% | AB306286.1 |
| Cold-inducible RNA-binding protein-like | comp361_c0_seq4 | 521 | *Anolis carolinensis* | 89% | XM_003224461.1 |
| Cold-inducible RNA-binding protein-like | comp3755_c0_seq1 | 577 | *Meleagris gallopavo* | 91% | XM_003205555.1 |
| Cold-inducible RNA-binding protein-like | comp1511_c0_seq1 | 855 | *Meleagris gallopavo* | 91% | XM_003205555.1 |
| Cold inducible RNA binding protein (CIRBP) | comp2385_c0_seq1 | 402 | *Gallus gallus* | 89% | NM_001031347.1 |
| **Sex-differentiation genes** |  |  |  |  |  |
| SOX2 | comp11084_c0_seq1 | 721 | *Danio rerio* | 83% | NP_998283.1 |
| SOX3 | comp21245_c0_seq1 | 654 | *Sus scrofa* | 69% | XP_003360512.1 |
| SOX4 | comp15705_c0_seq2 | 570 | *Nomascus leucogenys* | 92% | XP_003263605.1 |
| SOX4-2 | comp15705_c0_seq1 | 1214 | *Xenopus laevis* | 62% | AAI70171.1 |
| SOX5 | comp30944_c0_seq1 | 2115 | *Meleagris gallopavo* | 73% | XP_003202559.1 |
| SOX6 | comp287_c0_seq1 | 3530 | *Meleagris gallopavo* | 76% | XP_003206286.1 |
| SOX7 | comp23316_c0_seq1 | 632 | *Xenopus laevis* | 69% | AAI70531.1 |
| SOX8 | comp5283_c0_seq3 | 1592 | *Meleagris gallopavo* | 67% | XP_003210684.1 |
| SOX9 | comp5283_c0_seq4 | 1475 | *Pleurodeles waltl* | 84% | ACF95883.1 |
| SOX10 | comp46285_c0_seq1 | 582 | *Gallus gallus* | 78% | NP_990123.1 |
| SOX11 | comp45836_c0_seq1 | 278 | *Sus scrofa* | 78% | XP_003354985.1 |
| SOX13 | comp11526_c0_seq3 | 1321 | *Pan troglodytes* | 67% | XP_003308751.1 |
| SOX14 | comp19833_c0_seq1 | 700 | *Xenopus (Silurana) tropicalis* | 81% | NP_001093703.1 |
| SOX17 | comp113937_c0_seq1 | 475 | *Rattus norvegicus* | 61% | NP_001101372.1 |
| SOX18 | comp11526_c0_seq2 | 1435 | *Taeniopygia guttata* | 73% | XP_002197989.1 |
| SOX21 | comp67712_c0_seq1 | 471 | *Mus musculus* | 55% | NP_808421.1 |
| DMRTa1 | comp36581_c0_seq1 | 852 | *Monodelphis domestica* | 64% | XP_001374014.1 |
| DMRT2 | comp38891_c0_seq1 | 1082 | *Andrias davidianus* | 87% | FJ859987.1 |
| DMRT3 | comp134630_c0_seq1 | 306 | *Anolis carolinensis* | 88% | XM_003216487.1 |
| DMRT5 | comp102990_c0_seq1 | 466 | *Monopterus albus* | 83% | FJ455417.1 |
| WT1 | comp74827_c0_seq1 | 356 | *Cynops pyrrhogaster* | 85% | AB013888.1 |
| WNT4 | comp119465_c0_seq1 | 214 | *Xenopus (Silurana) tropicalis* | 100% | XP_002937081.1 |
| FOXL2 | comp6425_c0_seq6 | 247 | *Salmo salar* | 96% | HM159472.1 |
| **Reproduction related genes** |  |  |  |  |  |
| Degenerative spermatocyte homolog 3, lipid desaturase | comp7825_c1_seq1 | 2760 | *Xenopus (Silurana) tropicalis* | 83% | NP_988952.1 |
| Motile sperm domain containing 1 | comp17111_c0_seq1 | 440 | *Xenopus laevis* | 95% | NP_001080664.1 |
| Motile sperm domain containing 3 | comp5650_c0_seq1 | 4273 | *Xenopus (Silurana) tropicalis* | 60% | NP_001004905.1 |
| Novel protein similar to H.sapiens testis-specific kinase | comp84423_c0_seq1 | 201 | *Danio rerio* | 83% | CAX14101.1 |
| Oocyte antigenic protein | comp51051_c0_seq1 | 658 | *Xenopus laevis* | 68% | NP_001089128.1 |
| Oocyte-specific histone mRNA stem-loop binding protein | comp72358_c0_seq1 | 1398 | *Xenopus laevis* | 37% | AAI69951.1 |
| Outer dense fiber of sperm tails 2 | comp32766_c0_seq1 | 1847 | *Xenopus (Silurana) tropicalis* | 56% | NP_001098755.1 |
| Oocyte zinc finger protein XlCOF6-like | comp23148_c0_seq1 | 883 | *Danio rerio* | 67% | XP_003201615.1 |
| Similar to infertility-related sperm protein | comp4844_c0_seq1 | 2293 | *Gallus gallus* | 58% | XP_418360.2 |
| Similar to pregnancy-associated plasma protein A | comp84136_c0_seq1 | 368 | *Gallus gallus* | 89% | XP_415522.2 |
| Similar to pregnancy-induced growth inhibitor | comp111276_c0_seq1 | 329 | *Ornithorhynchus anatinus* | 89% | XP_001517136.1 |
| Similar to pregnancy-zone protein | comp96654_c0_seq1 | 231 | *Canis familiaris* | 50% | XP_854220.1 |
| Sex hormone-binding globulin | comp16339_c0_seq1 | 2461 | *Xenopus laevis* | 39% | NP_001088520.1 |
| Sperm associated antigen 6 | comp12690_c0_seq1 | 1718 | *Xenopus (Silurana) tropicalis* | 93% | NP_001015976.1 |
| Sperm associated antigen 9 | comp29949_c0_seq1 | 863 | *Bos taurus* | 81% | DAA18615.1 |
| Sperm flagellar protein 1 | comp67858_c0_seq1 | 320 | *Xenopus laevis* | 86% | NP_001090406.1 |
| Sperm flagellar protein 2 | comp26246_c0_seq1 | 772 | *Sus scrofa* | 68% | NP_001038026.1 |
| Spermatogenesis associated factor | comp90847_c0_seq1 | 474 | *Culex quinquefasciatus* | 96% | XP_001846323.1 |
| Spermatogenesis associated, serine-rich 2 | comp7617_c0_seq1 | 2332 | *Xenopus (Silurana) tropicalis* | 55% | NP_989244.1 |
| Spermatogenesis-associated protein 4 | comp14986_c0_seq1 | 1206 | *Oncorhynchus mykiss* | 59% | NP_001117998.1 |
| Spermatogenesis-associated protein 5-like protein 1 | comp75104_c0_seq1 | 230 | *Xenopus (Silurana) tropicalis* | 76% | NP_001072787.1 |
| Spermidine synthase | comp2741_c0_seq1 | 3144 | *Xenopus laevis* | 86% | NP_001084875.1 |
| Spermine oxidase | comp7593_c0_seq1 | 1989 | *Xenopus (Silurana) tropicalis* | 89% | NP_001039135.1 |
| Spermine synthase | comp65970_c0_seq1 | 328 | *Culex quinquefasciatus* | 76% | XP_001843925.1 |
| Testis expressed 2, gene 1 | comp120848_c0_seq1 | 320 | *Xenopus (Silurana) tropicalis* | 75% | NP_001188257.1 |
| Testis expressed 261 | comp2645_c0_seq1 | 2154 | *Xenopus (Silurana) tropicalis* | 92% | NP_001107323.1 |
| Testis specific, 10 | comp27140_c0_seq1 | 1374 | *Xenopus (Silurana) tropicalis* | 56% | NP_001120166.1 |
| Testis-specific protein pbs13 | comp129763_c0_seq1 | 208 | *Culex quinquefasciatus* | 76% | XP_001853447.1 |
| Vitellogenin | comp41321_c0_seq1 | 1224 | *Culex quinquefasciatus* | 48% | XP_001866876.1 |
| Vitellogenin 2 | comp29964_c0_seq1 | 388 | *Lepeophtheirus salmonis* | 35% | ABU41135.1 |
| Zona pellucida sperm-binding protein 1 | comp16955_c0_seq2 | 386 | *Gallus gallus* | 30% | NP_990014.1 |
