# Supplementary material for: Transcriptomic Analysis of Endangered Chinese Salamander: Identification of Immune, Sex and Reproduction-Related Genes and Genetic Markers
Source: PLoS One. 2014 Jan 31;9(1):e87940. doi: 10.1371/journal.pone.0087940 (PMC3909259; doi:10.1371/journal.pone.0087940)
Supplement: Table S6 — The 33 pairs of primer sequences used to assess the polymorphism of the putative cSSRs. (DOC) [file pone.0087940.s006.doc]

**Table S6** The 33 pairs of primer sequences used to assess the polymorphism of the putative cSSRs.

| **Unigenes** | | **Primer sequence** | |
| --- | --- | --- | --- |
| comp30707_c0_seq1 | | F:TATTCCTTTATTTAATGCTGTC | |
|  |  | R:GAGACGGATTCCCTTGAGTA | |
| comp5875_c0_seq1 | | F:TCATACACGGATTCATACAGA | |
|  |  | R:TGGAATAGACTTACGAATAAGAG | |
| comp77539_c0_seq1 | | F:AGCACTAAACCCATCCCAAT | |
|  |  | R:GCAGAGTCTGATGGGTGTATA | |
| comp5228_c0_seq3 | | F:ATCCAAGTTTGCCGTCAGAA | |
|  |  | R:AATCCGCCTCCTCGCTCT | |
| comp7499_c0_seq1 | | F:TCCTGTTAGTCTGAAAAGCC | |
|  |  | R:TCTCACGTAATGATTCTCCA | |
| comp16428_c0_seq1 | | F:ATGTCTATTGAGTGACTTGCT | |
|  |  | R:ATTGTAATGGTTTGGAGGAT | |
| comp35785_c0_seq1 | | F:TGTGACAGATGAGAATGAAACT | |
|  |  | R:TAGGGAAGGACAAAGCCA | |
| comp11916_c0_seq1 | | F:GCACTAGGGCACCCATAA | |
|  |  | R:TTTACGCTCTTTCCCCAA | |
| comp15080_c0_seq5 | | F:AGATGACAGAGGGGAGATTT | |
|  |  | R:GGCGGTGAGGAGGATGAGCG | |
| comp8900_c1_seq1 | | F:TCAATGGCATTATCAGTCC | |
|  |  | R:CCCCTTTTACACGGTCAC | |
| comp55574_c0_seq1 | | F:TACCTGGCTCTGTCCCT | |
|  |  | R:AACCTGCCTCTTTCCC | |
| comp38566_c0_seq1 | | F:GGAAAATCGCAGACACG | |
|  |  | R:CACATAGGAAATGACTCGG | |
| comp32386_c0_seq1 | | F:AAGCATTCCAGAAGGCG | |
|  |  | R:AACGGACCAGGGATACA | |
| comp17165_c0_seq4 | | F:CGGGCAAGCAAAACAAGG | |
|  |  | R:CCAGGGGTGGTGGGAGTT | |
| comp8202_c0_seq1 | | F:CTGGGACATACAGAGTGGGC | |
|  |  | R:CTCCCTCACAATGCTTCCTC | |
| comp10676_c0_seq1 | | F:AGGAACGCATACCTTGAT | |
|  |  | R:CTAGCCGTGGGAAAATAA | |
| comp5228_c0_seq3 | | F:ATCCAAGTTTGCCGTCAG | |
|  |  | R:AATCCGCCTCCTCGCTCT | |
| comp24606_c0_seq1 | | F:TCAAAAGGGCAGTTTATTC | |
|  |  | R:GTTGTGCTGTGATGTGGC | |
| comp1557_c0_seq1 | | F:GAGGAATGAACGGACGGG | |
|  |  | R:GTGGTGACTGGTTTTGCC | |
| comp4252_c0_seq1 | | F:GCCTTTCTAACCCTGCCGATA | |
|  |  | R:TCCGAGTGCCTTTGCTACAG | |
| comp11795_c0_seq2 | | F:GGAAGATTGTTGTGGGAGGG | |
|  |  | R:GCTACCAGGATGGCTTTCAA | |
| comp44630_c0_seq1 | | F:ACCCTCCATCATCTCACTTG | |
|  |  | R;GGGAAGCATCTGTTCTGTTG | |
| comp299_c2_seq11 | | F:CCTGTGATGATGGGAAGAAT | |
|  |  | R:GTGGTCAAACGTATCGTAGC | |
| comp454_c2_seq8 | | F:TCTTGCTGTGATTGTATGATT | |
|  |  | R:TGAACGGTCTACAGTCTACG | |
| comp454_c2_seq7 | | F:TTCCTGCTCTTGCTGTGATT | |
|  |  | R:TTTGATACATTGAACGGTCTA | |
| comp99364_c0_seq1 | | F:AGTGACTGAAGGGAGGCA | |
|  |  | R:GGTTAGCAGACCAGAGCAA | |
| comp45930_c0_seq1 | | F:GACTGGGAGGTCGCAT | |
|  |  | R:GAACAGGATAAACAGAGGG | |
| comp66054_c0_seq1 | | F:GCCAGAAATAGGGGTGA | |
|  |  | R:GCCAGGAACTCGTGATAG | |
| comp55641_c0_seq1 | | F:CCCTTTGGGGATTTGC | |
|  |  | R:CAGAGCCCTCCTCATACA | |
| comp31843_c0_seq1 | | F:CCTTTTAGGCTGTAGATGTT | |
|  |  | R:GGTCTGTCTCGTGGTCC | |
| comp13294_c0_seq1 | | F:GGGAGATGGGAACCGAT | |
|  |  | R:GCAGAAATGAACAGACCGT | |
| comp9937_c0_seq2 | | F:GTAGGTCAATGGGAGCAG | |
|  |  | R:GAGGAAATAATCCGAGCC | |
| comp80524_c0_seq1 | | F:GACATCACAATAGCCCTGC | |
|  | R:GTGCGGGTGTCAAGGTT | |  |

The first 13 pairs of primer sequences are these microsatellite loci that examined showing allelic polymorphism.
